# Supplementary material for: Ume6 protein complexes connect morphogenesis, adherence and hypoxic genes to shape Candida albicans biofilm architecture
Source: Nat Microbiol. 2025 Aug 21;10(9):2231–44. doi: 10.1038/s41564-025-02094-5 (PMC12408341; doi:10.1038/s41564-025-02094-5)
Supplement: Supplementary file 1 — Reporting Summary [file 41564_2025_2094_MOESM1_ESM.pdf]

nature portfolio

Corresponding author(s): Aaron P. Mitchell

Last updated by author(s): Jun 18, 2025

Reporting Summary

Nature Portfolio wishes to improve the reproducibility of the work that we publish. This form provides structure for consistency and transparency in reporting. For further information on Nature Portfolio policies, see our Editorial Policies and the Editorial Policy Checklist.

Statistics

For all statistical analyses, confirm that the following items are present in the figure legend, table legend, main text, or Methods section.

n/a	Confirmed	
☐	☒	The exact sample size (*n*) for each experimental group/condition, given as a discrete number and unit of measurement
☐	☒	A statement on whether measurements were taken from distinct samples or whether the same sample was measured repeatedly
☐	☒	The statistical test(s) used AND whether they are one- or two-sided<br>*Only common tests should be described solely by name; describe more complex techniques in the Methods section.*
☒	☐	A description of all covariates tested
☒	☐	A description of any assumptions or corrections, such as tests of normality and adjustment for multiple comparisons
☐	☒	A full description of the statistical parameters including central tendency (e.g. means) or other basic estimates (e.g. regression coefficient) AND variation (e.g. standard deviation) or associated estimates of uncertainty (e.g. confidence intervals)
☐	☒	For null hypothesis testing, the test statistic (e.g. *F*, *t*, *r*) with confidence intervals, effect sizes, degrees of freedom and *P* value noted<br>*Give P values as exact values whenever suitable.*
☒	☐	For Bayesian analysis, information on the choice of priors and Markov chain Monte Carlo settings
☒	☐	For hierarchical and complex designs, identification of the appropriate level for tests and full reporting of outcomes
☒	☐	Estimates of effect sizes (e.g. Cohen's *d*, Pearson's *r*), indicating how they were calculated

Our web collection on statistics for biologists contains articles on many of the points above.

Software and code

Policy information about availability of computer code

Data collection	Keyence BZ-X800 viewer was used for collection of microscopy data for biofilm assay.<br>AxioVision software (version 4.8.2.0) was used for collection of microscopy data for filamentation assay.<br>CFX Manager (version 3.1.1517.0823) was used for collection of both ChIP-qPCR and qRT-PCR data.
Data analysis	Details are included in the materials and methods section in the manuscript.<br><br>Fiji/ImageJ (ver2.14.0) was used for visualization and measurement of biofilm volume and hyphal cell length.<br>GraphPad Prism (ver9)<br>Mapping: hisat2 (ver 2.0.5)<br>Assembly: Stringtie (ver1.3.3b)<br>Quantification: featureCounts (ver1.5.0-p3)<br>Differential analysis: DESeq2 (ver1.22.1)<br>VolcaNoseR (https://huygens.science.uva.nl/VolcaNoseR2/)<br>IUPRED3 (https://iupred3.elte.hu/)

For manuscripts utilizing custom algorithms or software that are central to the research but not yet described in published literature, software must be made available to editors and reviewers. We strongly encourage code deposition in a community repository (e.g. GitHub). See the Nature Portfolio guidelines for submitting code & software for further information.

Data

Policy information about availability of data

All manuscripts must include a data availability statement. This statement should provide the following information, where applicable:

- Accession codes, unique identifiers, or web links for publicly available datasets
- A description of any restrictions on data availability
- For clinical datasets or third party data, please ensure that the statement adheres to our policy

Processed RNA-seq and ChIP-seq data are available in Table S1 and S2; raw data are available through NCBI SRA with accession numbers PRJNA1114072 (RNA-seq), PRJNA1114713 (Ume6-HA ChIP-seq), and PRJNA1114694 (Ume6-HA overexpression ChIP-seq).

Research involving human participants, their data, or biological material

Policy information about studies with human participants or human data. See also policy information about sex, gender (identity/presentation), and sexual orientation and race, ethnicity and racism.

Reporting on sex and gender	N/A
Reporting on race, ethnicity, or other socially relevant groupings	N/A
Population characteristics	N/A
Recruitment	N/A
Ethics oversight	N/A

Note that full information on the approval of the study protocol must also be provided in the manuscript.

Field-specific reporting

Please select the one below that is the best fit for your research. If you are not sure, read the appropriate sections before making your selection.

☒ Life sciences ☐ Behavioural & social sciences ☐ Ecological, evolutionary & environmental sciences

For a reference copy of the document with all sections, see nature.com/documents/nr-reporting-summary-flat.pdf

Life sciences study design

All studies must disclose on these points even when the disclosure is negative.

Sample size	No statistical methods were used to pre-determine sample sizes for any experiments in this study. For RNA-seq and ChIP-seq experiments, we employed three biological replicates. For biofilm assay, we employed three biological replicates. For ChIP-qPCR and qRT-PCR, we employed three biological replicates. For filamentation assay, we employed a hundred of hyphal cells from three independent images. For in vivo biofilm formation, we employed six biological replicates from two different experiments. These sample sizes are widely chosen for studies in our field.
Data exclusions	No data was excluded from analysis.
Replication	All experiments were subject to at least three biological replications unless otherwise stated.
Randomization	Randomization was not relevant for this study since samples were not allocated to experimental groups.
Blinding	Bilnding was not relevant for this study.

Reporting for specific materials, systems and methods

We require information from authors about some types of materials, experimental systems and methods used in many studies. Here, indicate whether each material, system or method listed is relevant to your study. If you are not sure if a list item applies to your research, read the appropriate section before selecting a response.

Materials & experimental systems

n/a	Involved in the study
☐	☒ Antibodies
☒	☐ Eukaryotic cell lines
☒	☐ Palaeontology and archaeology
☐	☒ Animals and other organisms
☒	☐ Clinical data
☒	☐ Dual use research of concern
☒	☐ Plants

Methods

n/a	Involved in the study
☐	☒ ChIP-seq
☒	☐ Flow cytometry
☒	☐ MRI-based neuroimaging

Antibodies

Antibodies used	Anti-HA (rabbit): polyclonal anti-HA tag antibody, abcam, catalog # ab9110, 7ul used for ChIP, 1ul used for Co-IP, 1:10000 dilution for Western blotting.<br>Anti-FLAG (mouse): monoclonal anti-FLAG M2 antibody, Sigma, catalog #F3165, 1:5000 dilution for Western blotting.<br>Goat anti-mouse: anti-Mouse IgG (H+L) HRP from goat, ThermoFisher, Catalog # 31430, 1:5000 dilution for Western blotting.<br>Goat anti-rabbit: anti-rabbit IgG-HRP from goat, Jackson ImmunoResearch, catalog # 111-035-144, 1:5000 dilution for Western blotting.
Validation	All anti-bodies used in this manuscript was validated in our previous publication.<br>Do E, Cravener MV, Huang MY, May G, McManus CJ, Mitchell AP. Collaboration between Antagonistic Cell Type Regulators Governs Natural Variation in the Candida albicans Biofilm and Hyphal Gene Expression Network. mBio. 2022 Oct 26;13(5):e0193722. doi: 10.1128/mbio.01937-22. Epub 2022 Aug 22. PMID: 35993746; PMCID: PMC9600859.

Animals and other research organisms

Policy information about studies involving animals; ARRIVE guidelines recommended for reporting animal research, and Sex and Gender in Research

Laboratory animals	Sprague Dawley rats, 16-week old, 400 g
Wild animals	Wild animals were not used in this study.
Reporting on sex	Male mice were used for experiments.
Field-collected samples	The studies did not involve field collected samples.
Ethics oversight	All animal procedures were approved by the Institutional Animal Care and Use Committee at the University of Wisconsin-Madison according to the guidelines of the Animal Welfare Act, The Institute of Laboratory Animal Resources Guide for the care and Use of Laboratory Animals, and Public Health Service Policy. The approved animal protocol number is DA0031.

Note that full information on the approval of the study protocol must also be provided in the manuscript.

Plants

Seed stocks	N/A
Novel plant genotypes	N/A
Authentication	N/A

ChIP-seq

Data deposition

☒ Confirm that both raw and final processed data have been deposited in a public database such as GEO.

☒ Confirm that you have deposited or provided access to graph files (e.g. BED files) for the called peaks.

Data access links
May remain private before publication.

www.ncbi.nlm.nih.gov/bioproject/PRJNA1114713
www.ncbi.nlm.nih.gov/bioproject/PRJNA1114694
All processed data has been submitted in the supplemental data.

Files in database submission

ED1_R_S1_L001_R1_001.fastq.gz
ED2_R_S2_L001_R1_001.fastq.gz
ED3_R_S3_L001_R1_001.fastq.gz
ED4_R_S4_L001_R1_001.fastq.gz
ED5_R_S5_L001_R1_001.fastq.gz
ED6_R_S6_L001_R1_001.fastq.gz
ED7_R_S7_L001_R1_001.fastq.gz
ED8_R_S8_L001_R1_001.fastq.gz
ED9_R_S9_L001_R1_001.fastq.gz
ED10_R_S10_L001_R1_001.fastq.gz
ED11_R_S11_L001_R1_001.fastq.gz
ED12_R_S12_L001_R1_001.fastq.gz
ED13_R_S13_L001_R1_001.fastq.gz
ED14_R_S14_L001_R1_001.fastq.gz
ED15_R_S15_L001_R1_001.fastq.gz
ED16_R_S16_L001_R1_001.fastq.gz
ED17_R_S17_L001_R1_001.fastq.gz
ED18_R_S18_L001_R1_001.fastq.gz
ED19_R_S19_L001_R1_001.fastq.gz
ED20_R_S20_L001_R1_001.fastq.gz
ED21_R_S21_L001_R1_001.fastq.gz
ED22_R_S22_L001_R1_001.fastq.gz
ED1_R_S1_L001_R2_001.fastq.gz
ED2_R_S2_L001_R2_001.fastq.gz
ED3_R_S3_L001_R2_001.fastq.gz
ED4_R_S4_L001_R2_001.fastq.gz
ED5_R_S5_L001_R2_001.fastq.gz
ED6_R_S6_L001_R2_001.fastq.gz
ED7_R_S7_L001_R2_001.fastq.gz
ED8_R_S8_L001_R2_001.fastq.gz
ED9_R_S9_L001_R2_001.fastq.gz
ED10_R_S10_L001_R2_001.fastq.gz
ED11_R_S11_L001_R2_001.fastq.gz
ED12_R_S12_L001_R2_001.fastq.gz
ED13_R_S13_L001_R2_001.fastq.gz
ED14_R_S14_L001_R2_001.fastq.gz
ED15_R_S15_L001_R2_001.fastq.gz
ED16_R_S16_L001_R2_001.fastq.gz
ED17_R_S17_L001_R2_001.fastq.gz
ED18_R_S18_L001_R2_001.fastq.gz
ED19_R_S19_L001_R2_001.fastq.gz
ED20_R_S20_L001_R2_001.fastq.gz
ED21_R_S21_L001_R2_001.fastq.gz
ED22_R_S22_L001_R2_001.fastq.gz
ED1_4349_S1_L001_R1_001.fastq.gz
ED2_4349_S1_L001_R1_001.fastq.gz
ED3_4349_S2_L001_R1_001.fastq.gz
ED4_4349_S2_L001_R1_001.fastq.gz
ED5_4349_S3_L001_R1_001.fastq.gz
ED6_4349_S3_L001_R1_001.fastq.gz
ED7_4349_S4_L001_R1_001.fastq.gz
ED8_4349_S4_L001_R1_001.fastq.gz
ED9_4349_S5_L001_R1_001.fastq.gz
ED16_4349_S8_L001_R1_001.fastq.gz
ED17_4349_S9_L001_R1_001.fastq.gz
ED18_4349_S9_L001_R1_001.fastq.gz
ED19_4349_S10_L001_R1_001.fastq.gz
ED20_4349_S10_L001_R1_001.fastq.gz
ED1_4349_S1_L001_R2_001.fastq.gz
ED2_4349_S1_L001_R2_001.fastq.gz
ED3_4349_S2_L001_R2_001.fastq.gz
ED4_4349_S2_L001_R2_001.fastq.gz
ED5_4349_S3_L001_R2_001.fastq.gz
ED6_4349_S3_L001_R2_001.fastq.gz
ED7_4349_S4_L001_R2_001.fastq.gz
ED8_4349_S4_L001_R2_001.fastq.gz
ED9_4349_S5_L001_R2_001.fastq.gz
ED16_4349_S8_L001_R2_001.fastq.gz
ED17_4349_S9_L001_R2_001.fastq.gz
ED18_4349_S9_L001_R2_001.fastq.gz

	ED19_4349_S10_L001_R2_001.fastq.gz<br>ED20_4349_S10_L001_R2_001.fastq.gz
Genome browser session (e.g. UCSC)	Not applicable

Methodology

Replicates	All ChIP-seq experiments was performed with biological triplicate samples.
Sequencing depth	ChIP-seq libraries were sequenced on Illumina NextSeq 2000 sequencing system (Illumina, 2 x 51 nt, paired-end).<br>Raw read-depth ranged from 24 M to 65 M read pairs per sample. To normalize read depth, we downsampled the number of aligned reads to 5 M pairs (10 M total reads) for each sample before calling peaks.
Antibodies	Anti-HA rabbit polyclonal (abcam: ab9110) was used for ChIP-seq experiments.
Peak calling parameters	MACS2 (v 2.1.0.20151222; default parameters.)
Data quality	Threshold of False discovery rate (FDR) was set at 0.05 and the peaks consistent across the replicates were further used in the annotation.
Software	bowtie2 (v 2.1.0)<br>samtools (v1.10)<br>MACS2 (v 2.1.0.20151222)<br>mspc (v 5.4.0)<br>DiffBind (Galaxy Version 2.10.0+galaxy0)<br>HOMER (v 4.11)<br>Integrative Genomics Viewer v2.11.0
