# Supplementary material for: Ume6 protein complexes connect morphogenesis, adherence and hypoxic genes to shape Candida albicans biofilm architecture
Source: Nat Microbiol. 2025 Aug 21;10(9):2231–44. doi: 10.1038/s41564-025-02094-5 (PMC12408341; doi:10.1038/s41564-025-02094-5)
Supplement: Supplementary file 9 — Unprocessed western blots. [file 41564_2025_2094_MOESM9_ESM.pdf]

Fig 1a

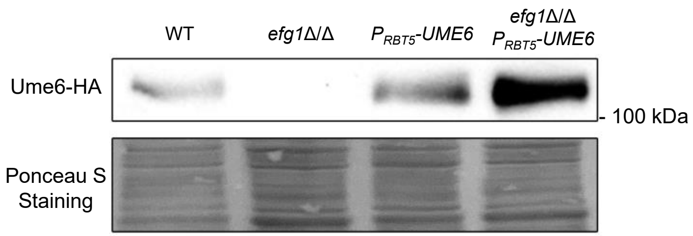

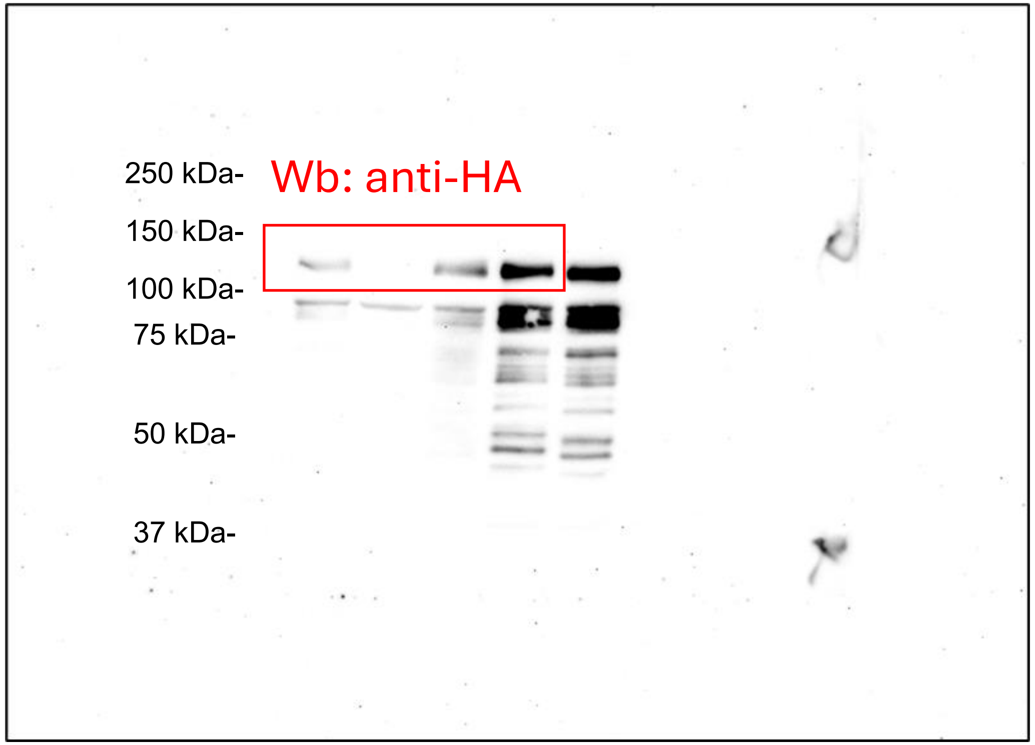

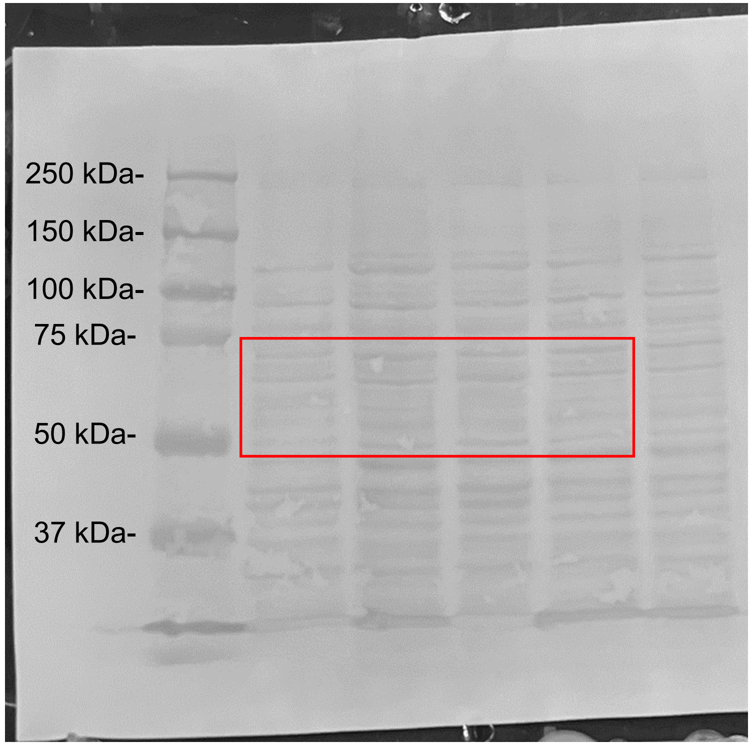

Wb: anti-HA

Membrane stained with Ponceau S

Fig 3c

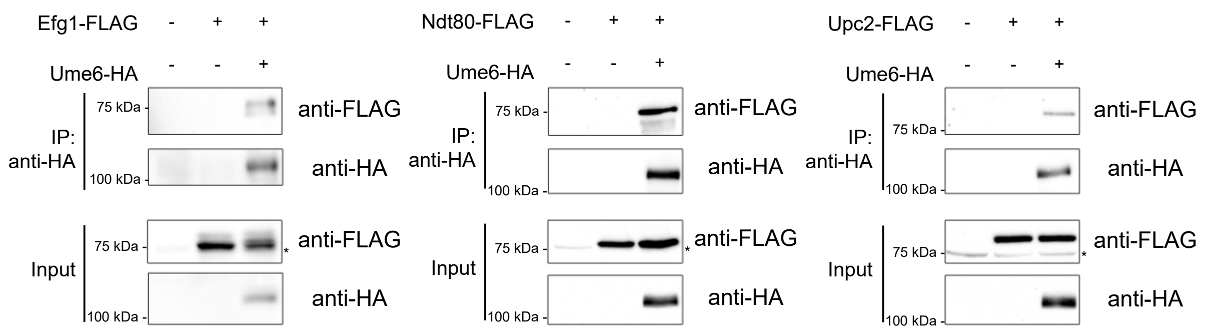
Efg1-FLAG - + +
Ume6-HA - - +
IP: anti-HA
75 kDa
anti-FLAG
100 kDa
anti-HA
Input
75 kDa
anti-FLAG
100 kDa
anti-HA
Ndt80-FLAG - + +
Ume6-HA - - +
IP: anti-HA
75 kDa
anti-FLAG
100 kDa
anti-HA
Input
75 kDa
anti-FLAG
100 kDa
anti-HA
Upc2-FLAG - + +
Ume6-HA - - +
IP: anti-HA
75 kDa
anti-FLAG
100 kDa
anti-HA
Input
75 kDa
anti-FLAG
100 kDa
anti-HA

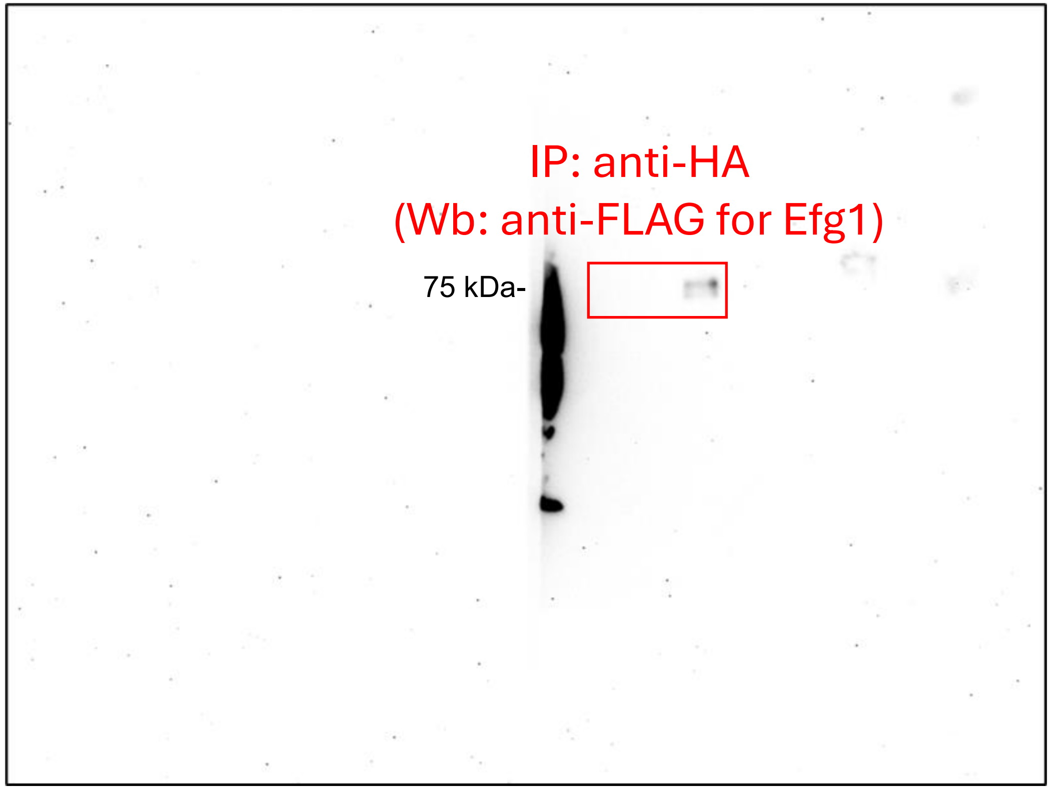
IP: anti-HA
(Wb: anti-FLAG for Efg1)
75 kDa-

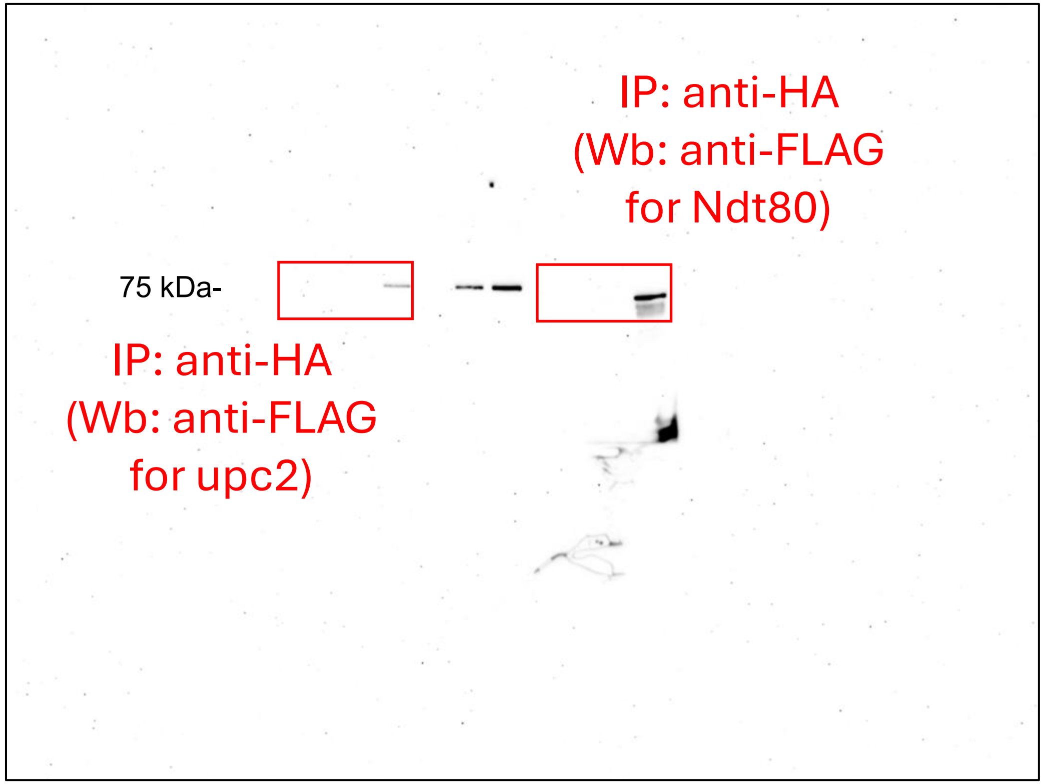
IP: anti-HA
(Wb: anti-FLAG
for Ndt80)
75 kDa-
IP: anti-HA
(Wb: anti-FLAG
for upc2)

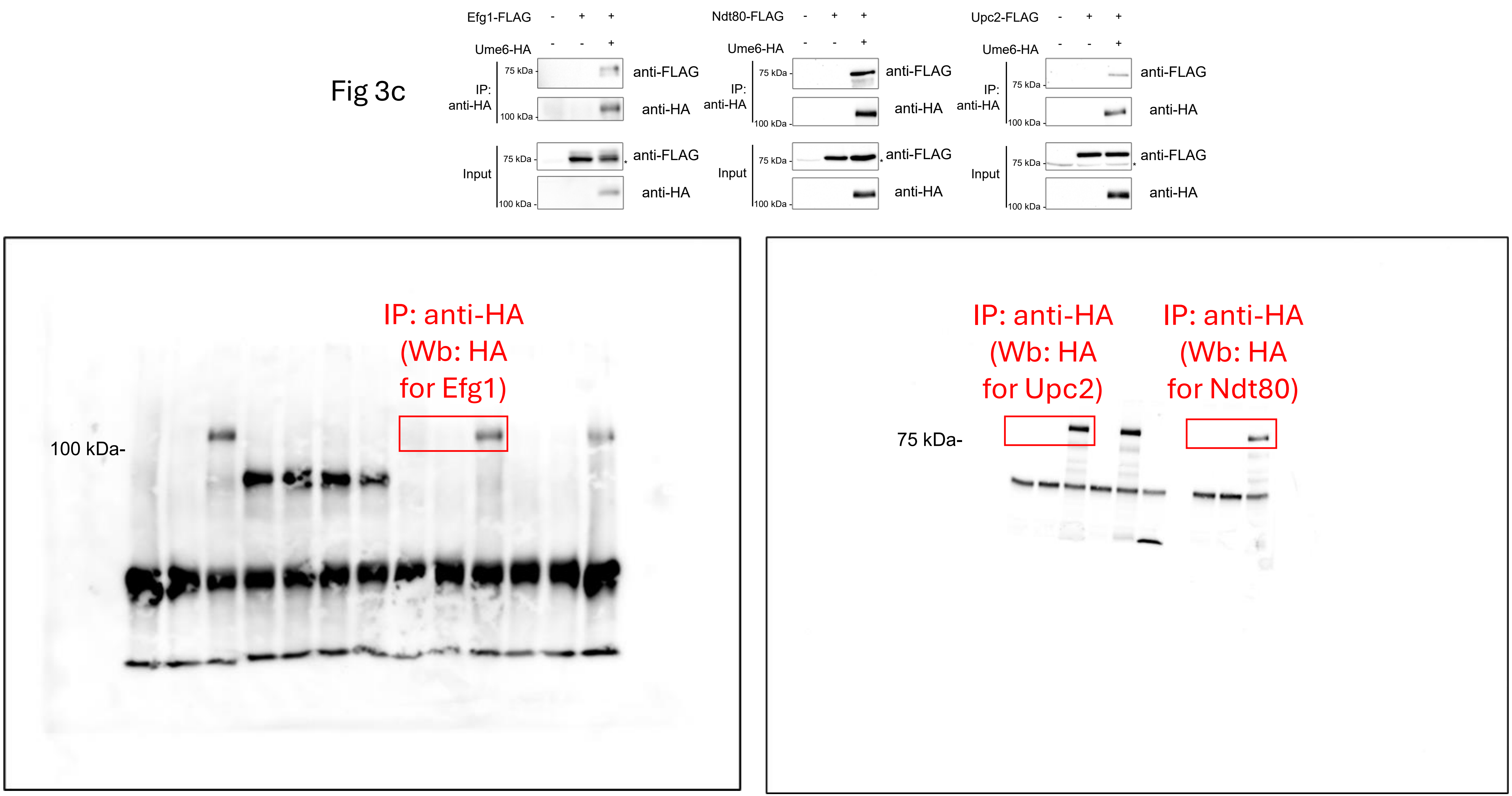
Fig 3c
Efg1-FLAG - + +
Ume6-HA - - +
IP: anti-HA
75 kDa
100 kDa
anti-FLAG
anti-HA
Input
Ndt80-FLAG - + +
Ume6-HA - - +
Upc2-FLAG - + +
Ume6-HA - - +
IP: anti-HA (Wb: HA for Efg1)
100 kDa-
IP: anti-HA (Wb: HA for Upc2)
IP: anti-HA (Wb: HA for Ndt80)
75 kDa-

Fig 3c

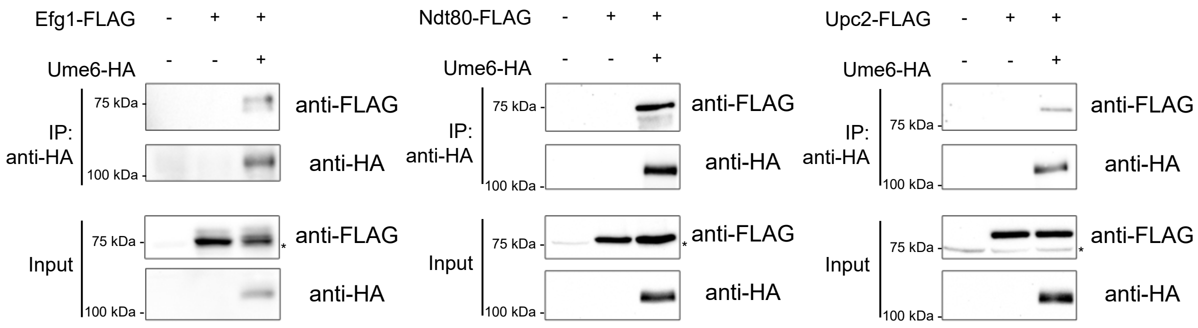
Efg1-FLAG - + +
Ume6-HA - - +
IP: anti-HA
75 kDa
100 kDa
anti-FLAG
anti-HA
Input
anti-FLAG
anti-HA
Ndt80-FLAG - + +
Ume6-HA - - +
Upc2-FLAG - + +
Ume6-HA - - +

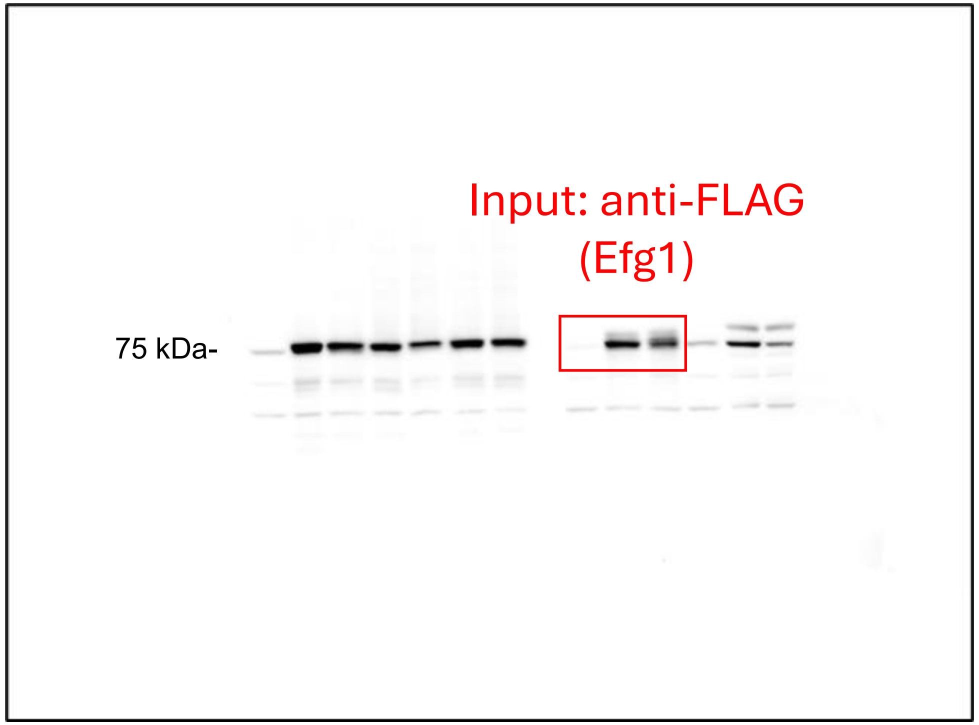
Input: anti-FLAG
(Efg1)
75 kDa-

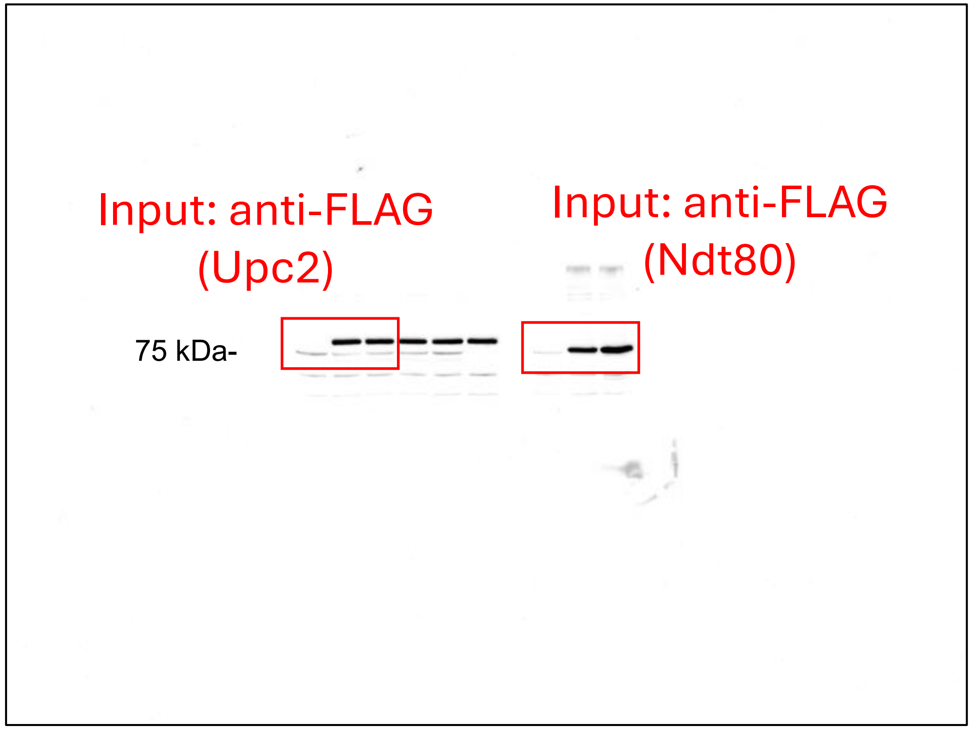
Input: anti-FLAG
(Upc2)
Input: anti-FLAG
(Ndt80)
75 kDa-

Fig 3c

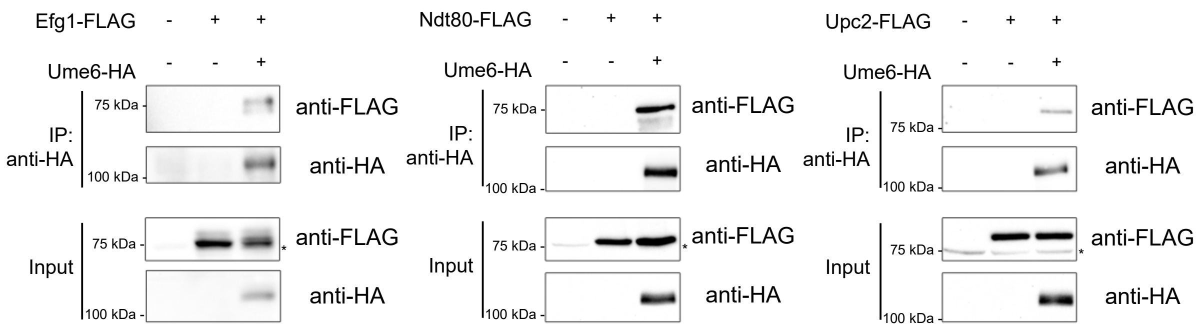
Efg1-FLAG - + +
Ume6-HA - - +
IP: anti-HA
75 kDa
100 kDa
anti-FLAG
anti-HA
Input
75 kDa
100 kDa
anti-FLAG
anti-HA
Ndt80-FLAG - + +
Ume6-HA - - +
IP: anti-HA
75 kDa
100 kDa
anti-FLAG
anti-HA
Input
75 kDa
100 kDa
anti-FLAG
anti-HA
Upc2-FLAG - + +
Ume6-HA - - +
IP: anti-HA
75 kDa
100 kDa
anti-FLAG
anti-HA
Input
75 kDa
100 kDa
anti-FLAG
anti-HA

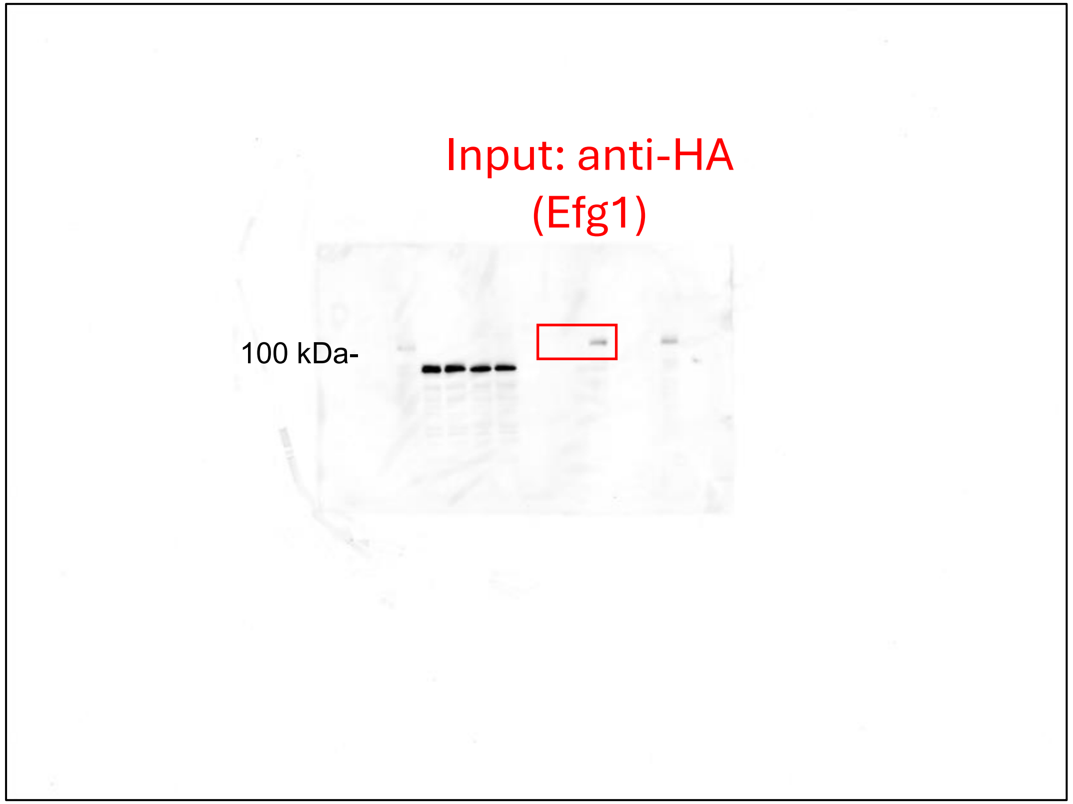
Input: anti-HA
(Efg1)
100 kDa-

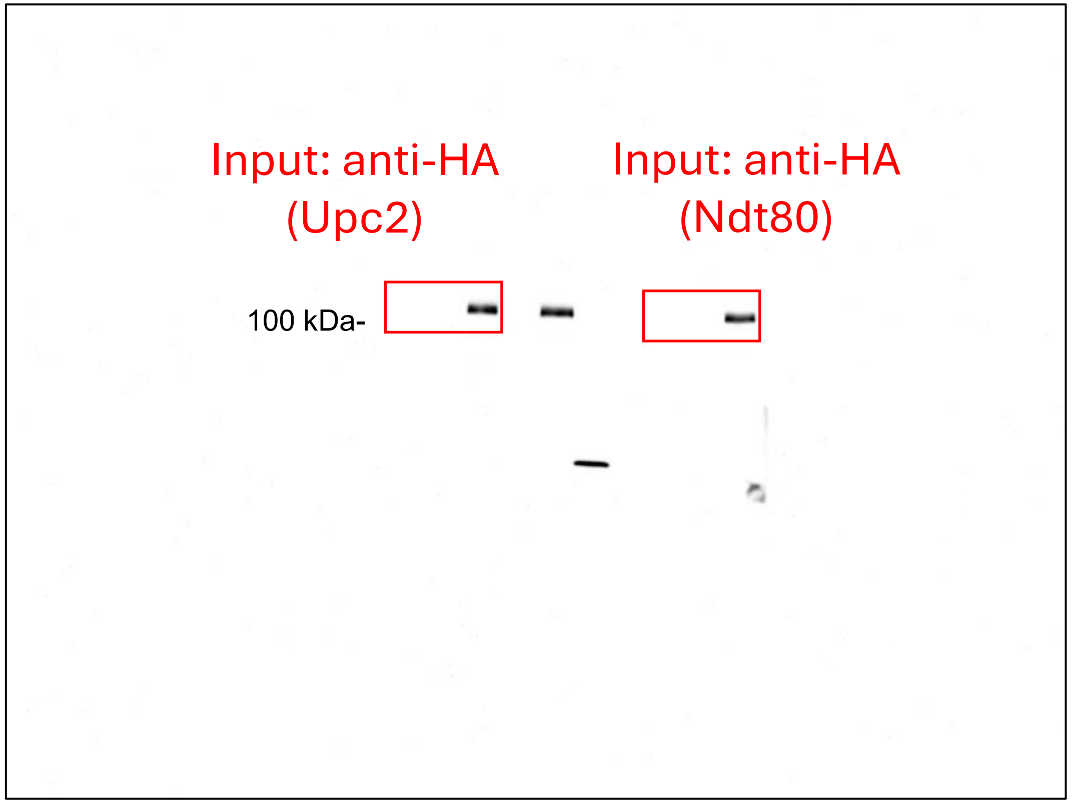
Input: anti-HA
(Upc2)
Input: anti-HA
(Ndt80)
100 kDa-

Fig 3d

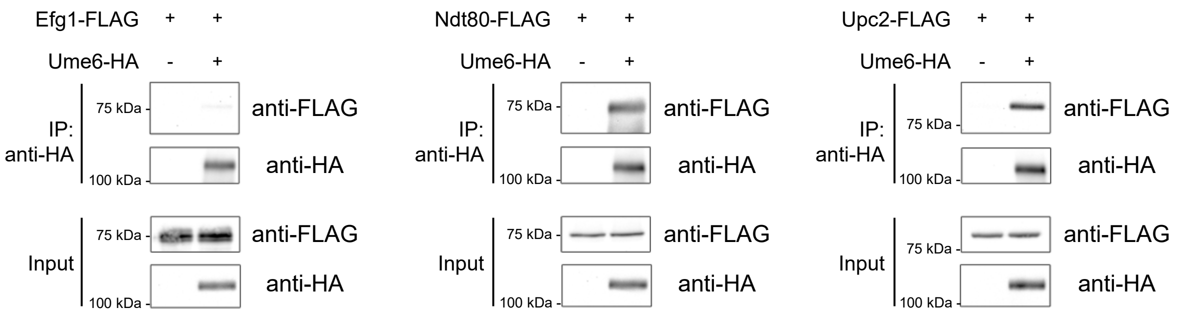

Efg1-FLAG + +
Ume6-HA - +
IP: anti-HA
75 kDa
anti-FLAG
100 kDa
anti-HA
Input
75 kDa
anti-FLAG
100 kDa
anti-HA
Ndt80-FLAG + +
Ume6-HA - +
IP: anti-HA
75 kDa
anti-FLAG
100 kDa
anti-HA
Input
75 kDa
anti-FLAG
100 kDa
anti-HA
Upc2-FLAG + +
Ume6-HA - +
IP: anti-HA
75 kDa
anti-FLAG
100 kDa
anti-HA
Input
75 kDa
anti-FLAG
100 kDa
anti-HA

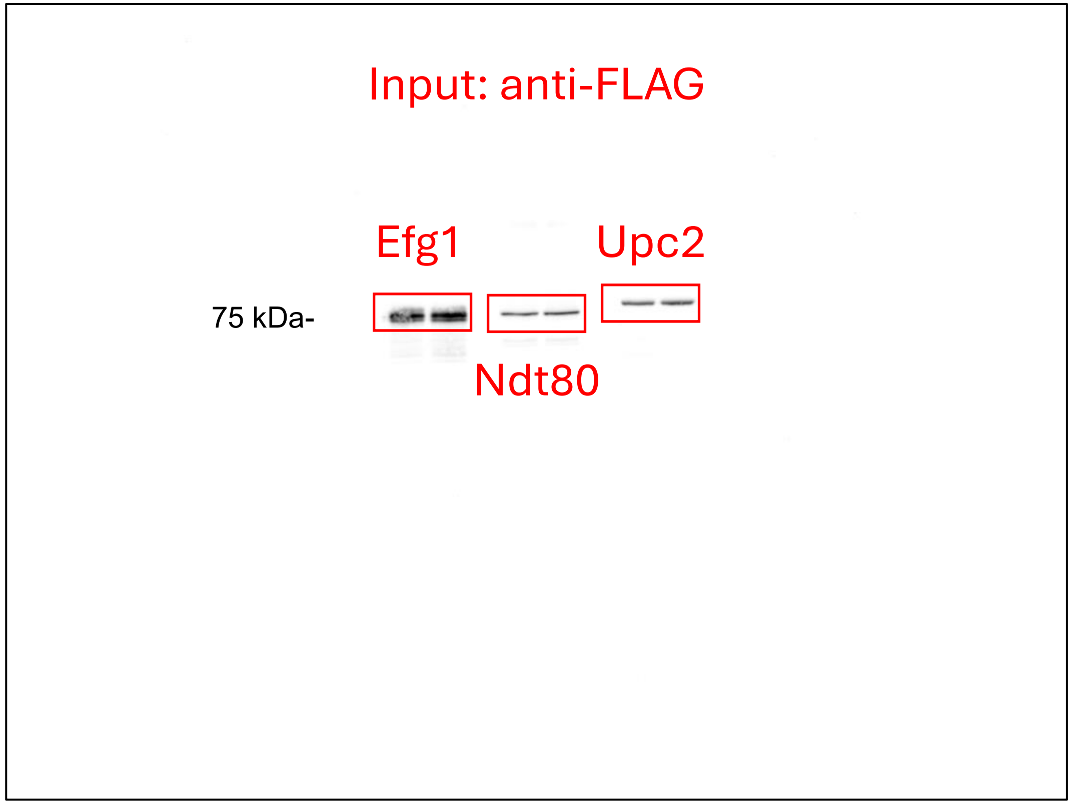

Input: anti-FLAG
Efg1
Upc2
75 kDa-
Ndt80

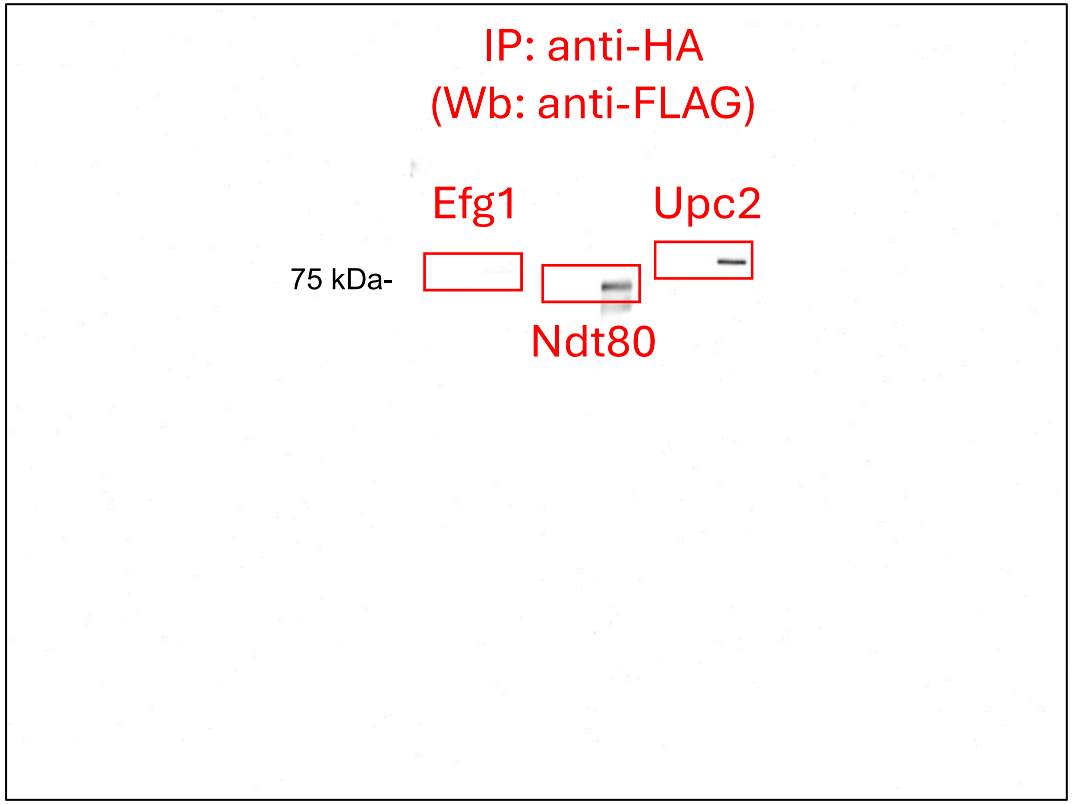

IP: anti-HA
(Wb: anti-FLAG)
Efg1
Upc2
75 kDa-
Ndt80

Fig 3d

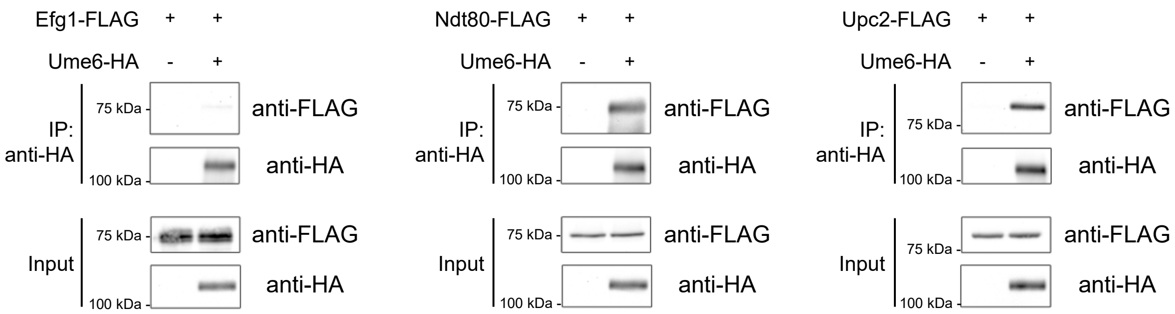

Efg1-FLAG + +
Ume6-HA - +
IP: anti-HA
75 kDa
100 kDa
anti-FLAG
anti-HA
Input
Ndt80-FLAG + +
Ume6-HA - +
Upc2-FLAG + +
Ume6-HA - +

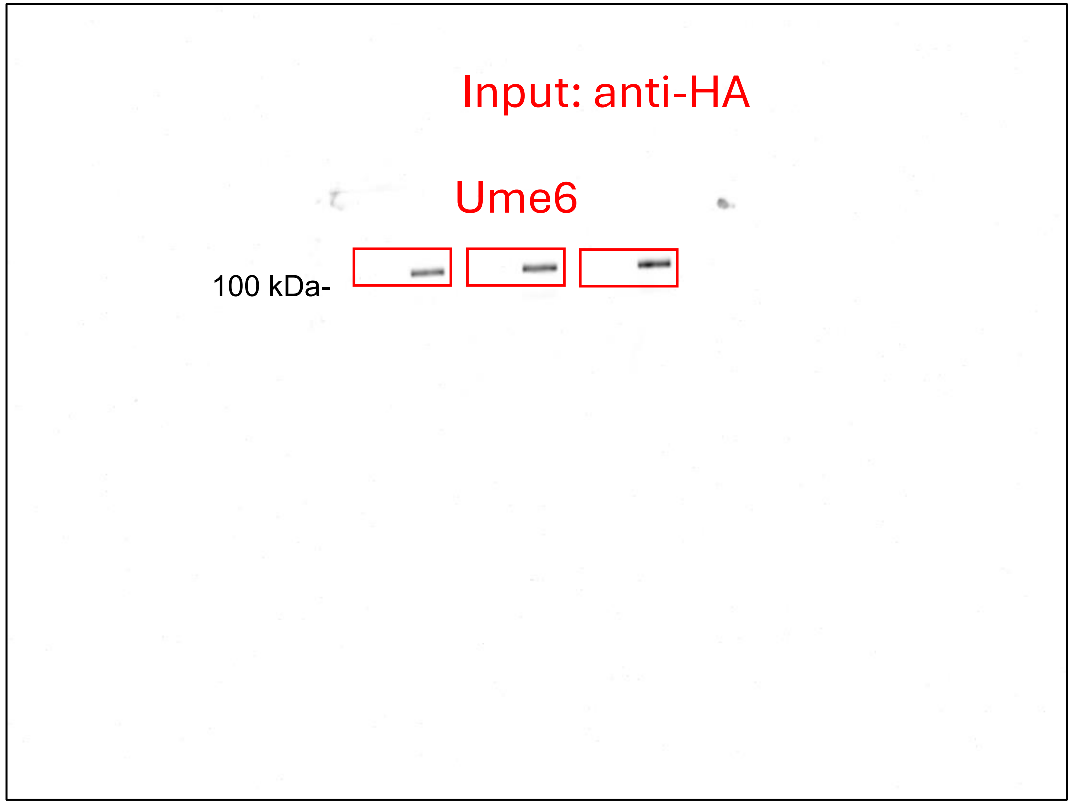

Input: anti-HA
Ume6
100 kDa-

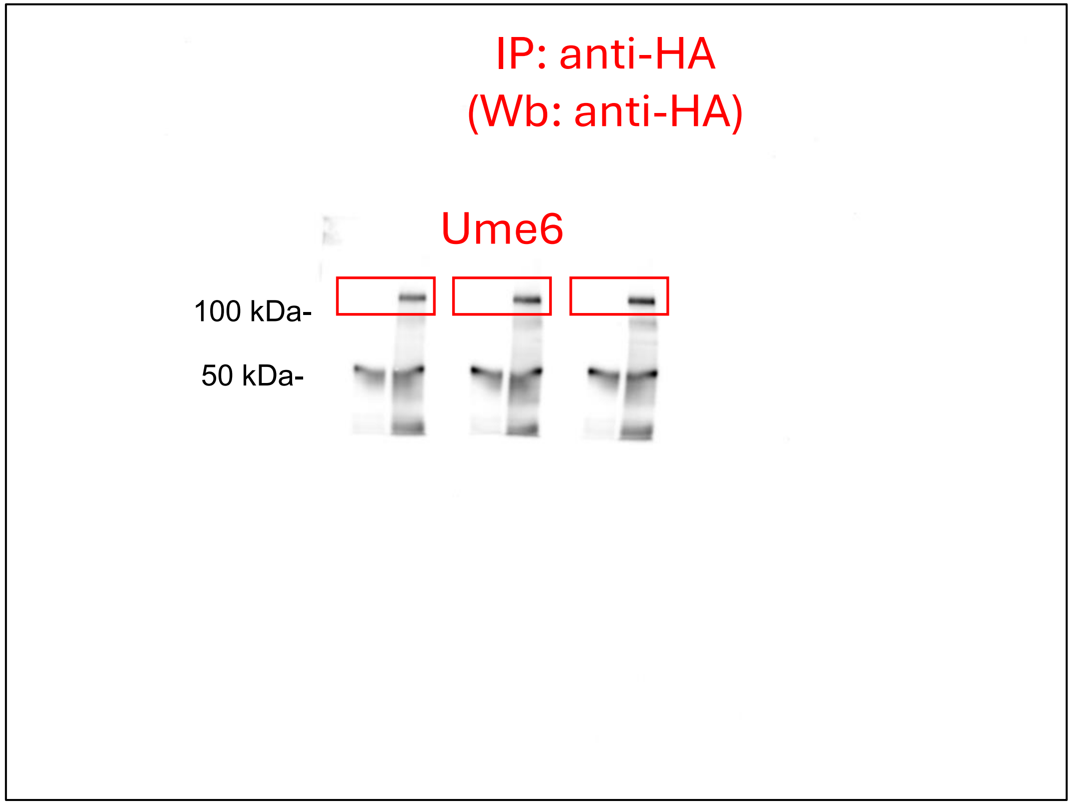

IP: anti-HA
(Wb: anti-HA)
Ume6
100 kDa-
50 kDa-

Fig 4d

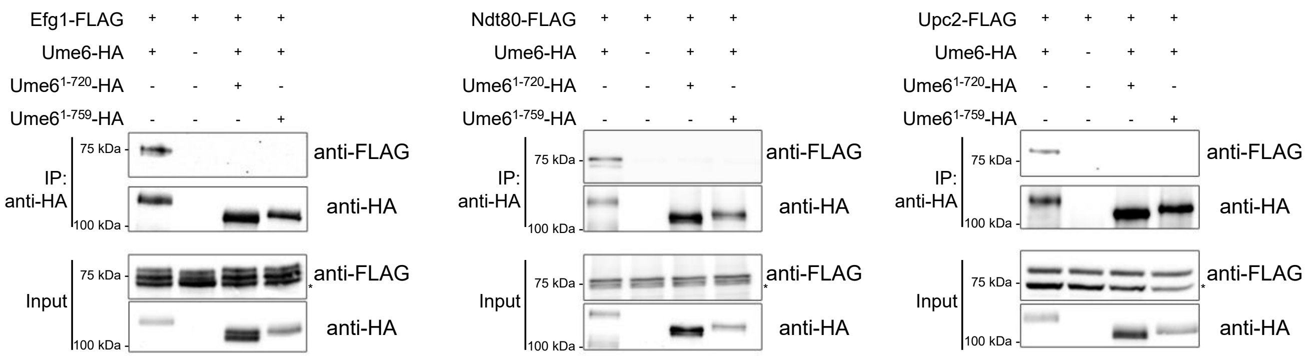
Efg1-FLAG + + + +
Ume6-HA + - + +
$Ume6^{1-720}$-HA - - + -
$Ume6^{1-759}$-HA - - - +
IP: anti-HA
Input
75 kDa
100 kDa
anti-FLAG
anti-HA
Ndt80-FLAG + + + +
Ume6-HA + - + +
$Ume6^{1-720}$-HA - - + -
$Ume6^{1-759}$-HA - - - +
Upc2-FLAG + + + +
Ume6-HA + - + +
$Ume6^{1-720}$-HA - - + -
$Ume6^{1-759}$-HA - - - +

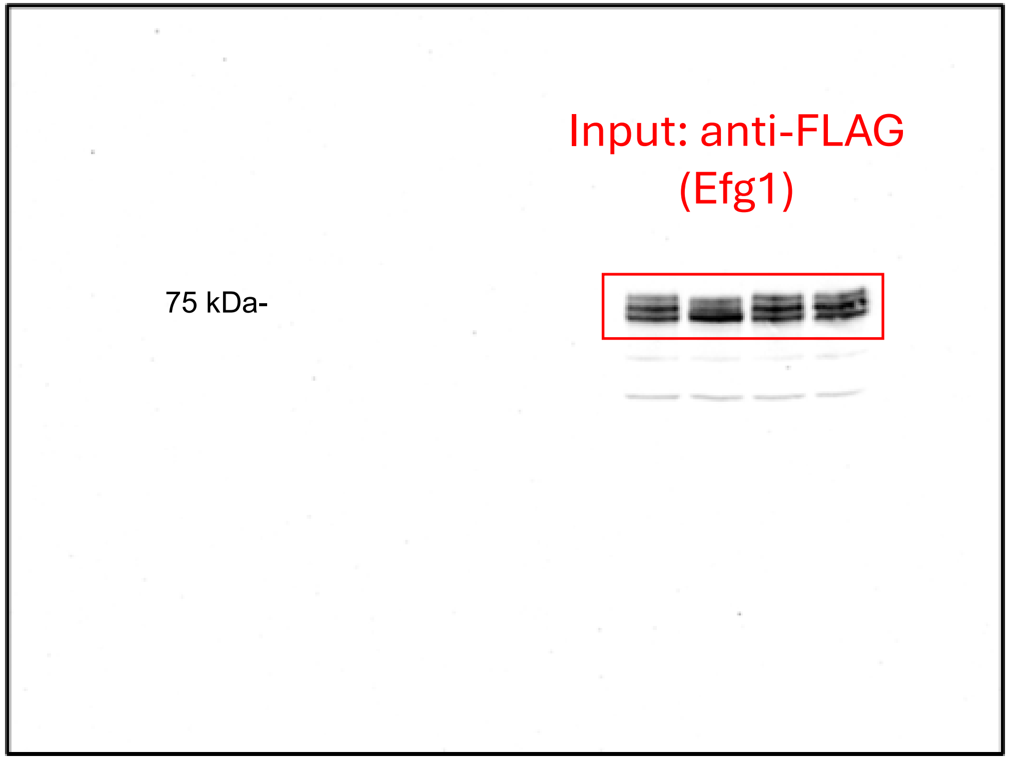
Input: anti-FLAG
(Efg1)
75 kDa-

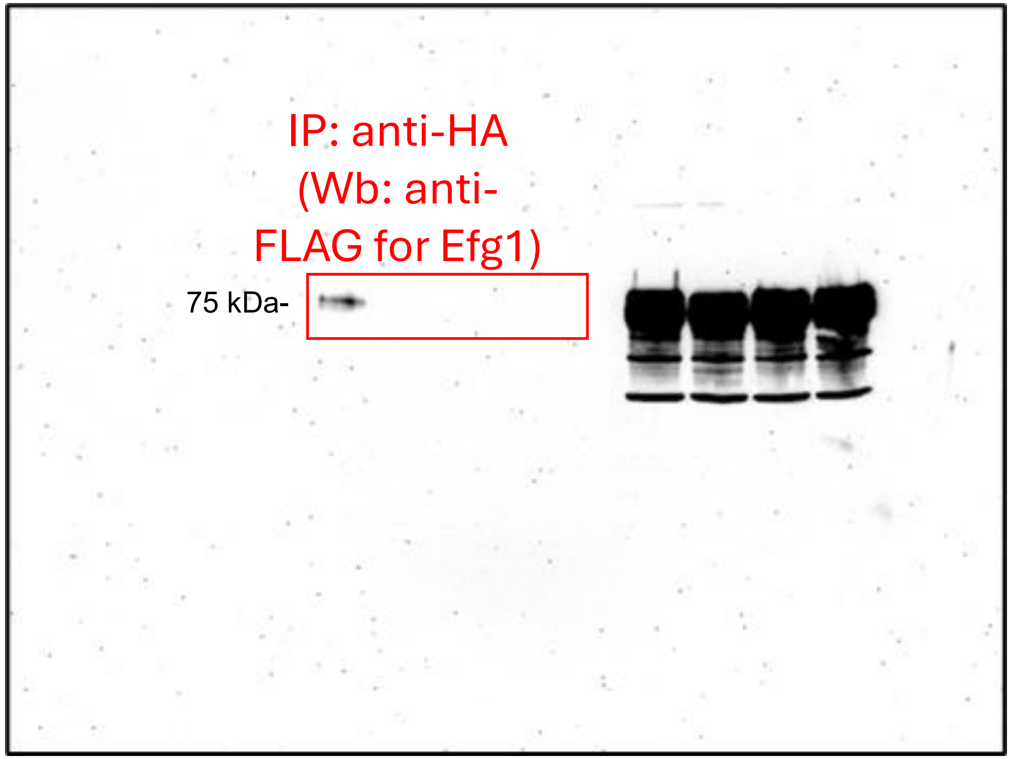
IP: anti-HA
(Wb: anti-
FLAG for Efg1)
75 kDa-

Fig 4d

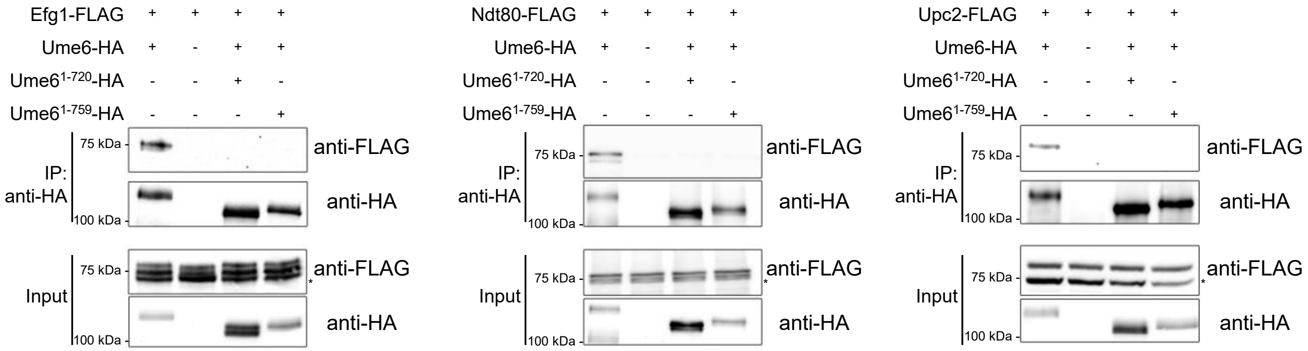
Efg1-FLAG + + + +
Ume6-HA + - + +
Ume6$^{1-720}$-HA - - + -
Ume6$^{1-759}$-HA - - - +
Ndt80-FLAG + + + +
Ume6-HA + - + +
Ume6$^{1-720}$-HA - - + -
Ume6$^{1-759}$-HA - - - +
Upc2-FLAG + + + +
Ume6-HA + - + +
Ume6$^{1-720}$-HA - - + -
Ume6$^{1-759}$-HA - - - +
IP: anti-HA
Input
75 kDa
100 kDa
anti-FLAG
anti-HA

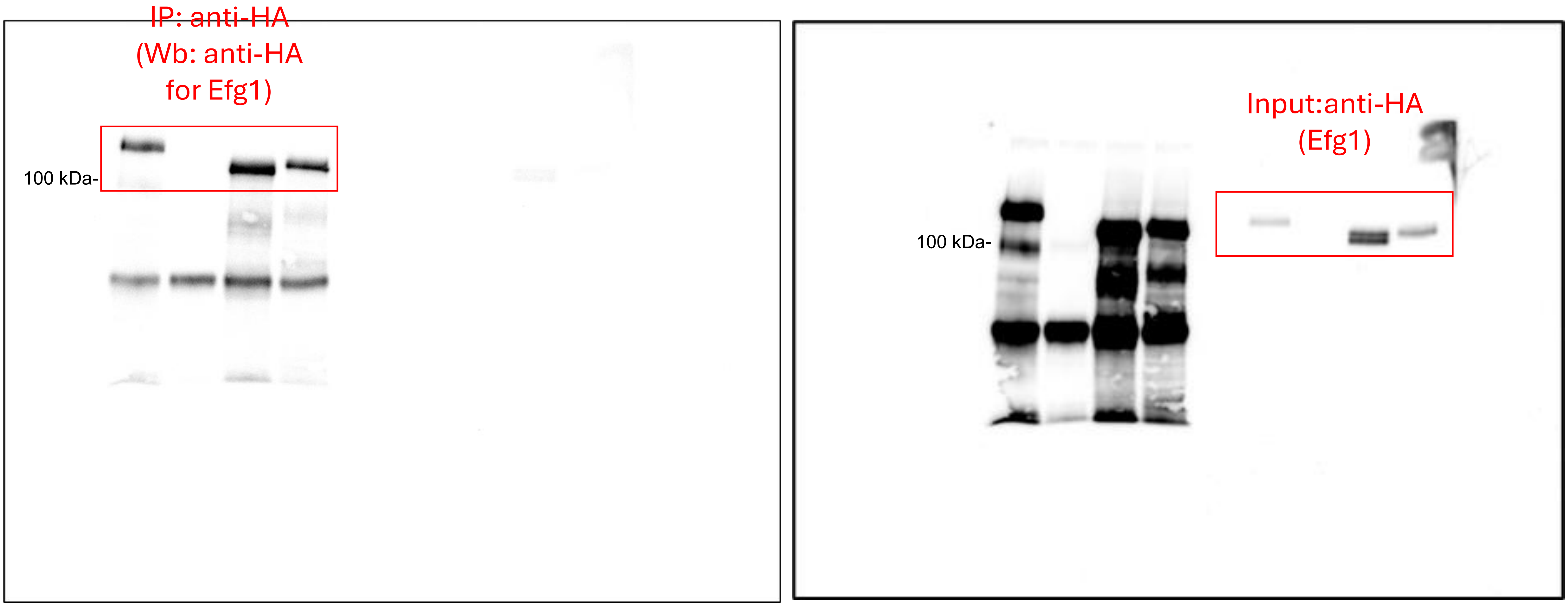
IP: anti-HA
(Wb: anti-HA
for Efg1)
100 kDa-
Input:anti-HA
(Efg1)
100 kDa-

Fig 4d

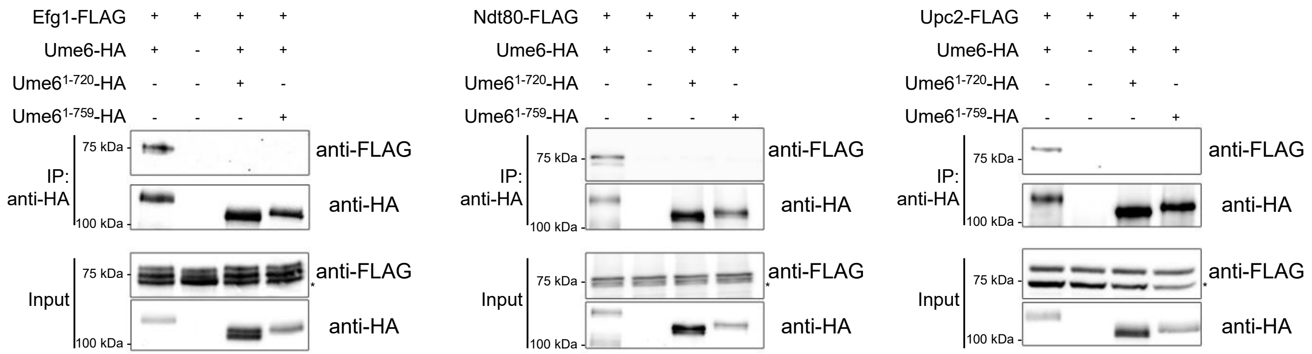
Efg1-FLAG + + + +
Ume6-HA + - + +
Ume6$^{1-720}$-HA - - + -
Ume6$^{1-759}$-HA - - - +
Ndt80-FLAG + + + +
Ume6-HA + - + +
Ume6$^{1-720}$-HA - - + -
Ume6$^{1-759}$-HA - - - +
Upc2-FLAG + + + +
Ume6-HA + - + +
Ume6$^{1-720}$-HA - - + -
Ume6$^{1-759}$-HA - - - +
IP: anti-HA
Input
75 kDa
100 kDa
anti-FLAG
anti-HA

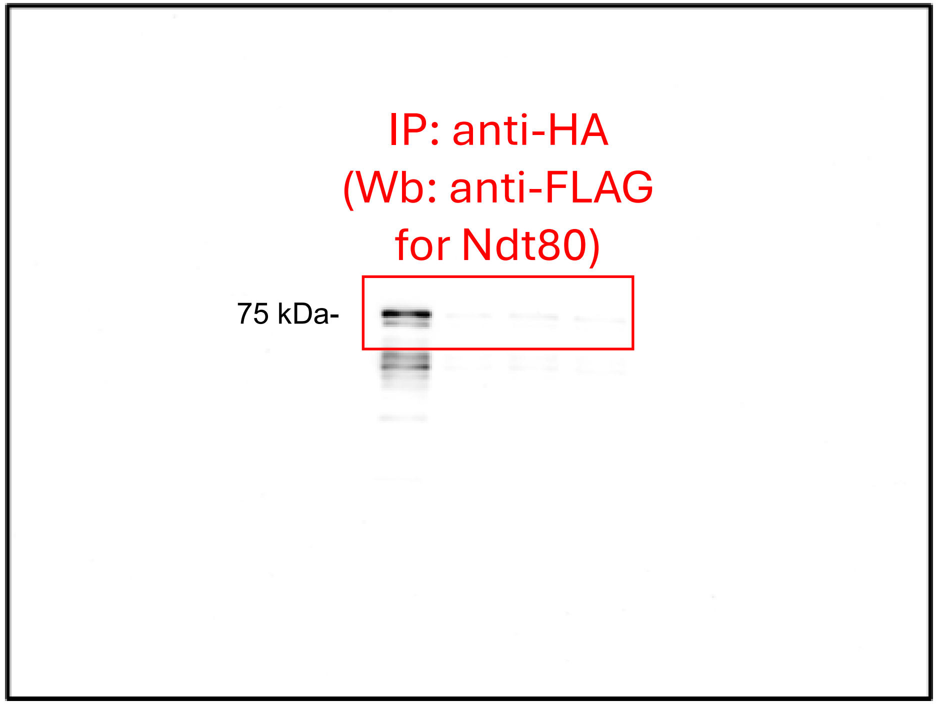
IP: anti-HA
(Wb: anti-FLAG
for Ndt80)
75 kDa-

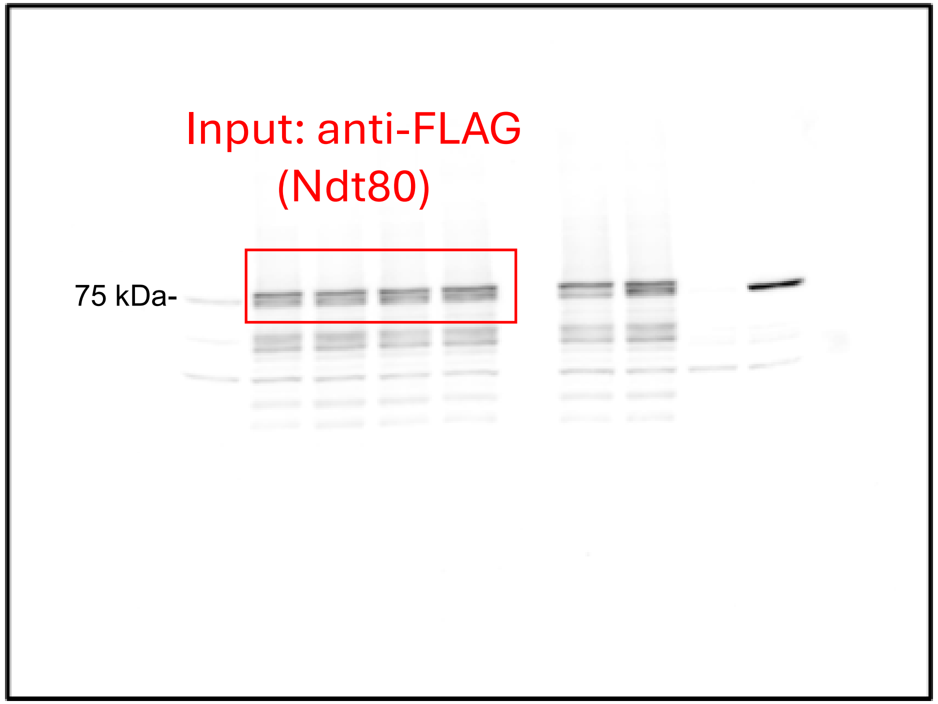
Input: anti-FLAG
(Ndt80)
75 kDa-

Fig 4d

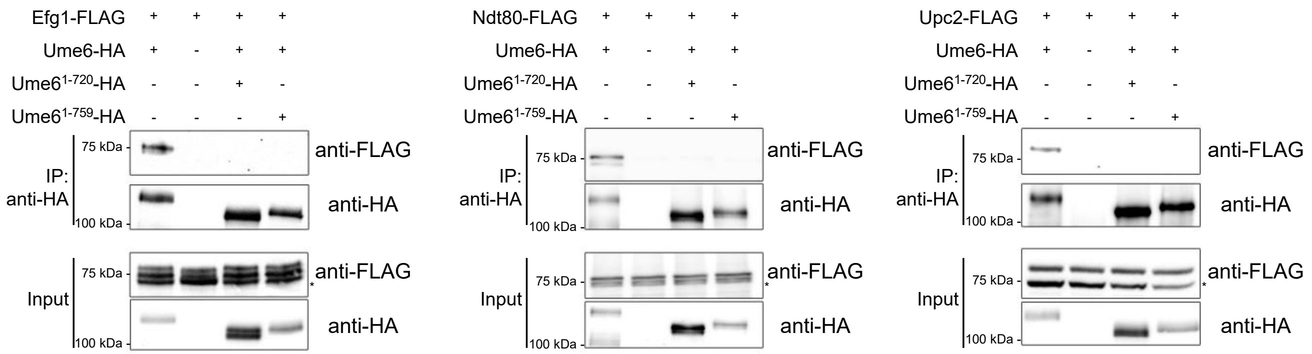
Efg1-FLAG + + + +
Ume6-HA + - + +
Ume6^1-720-HA - - + -
Ume6^1-759-HA - - - +
IP: anti-HA
Input
75 kDa
100 kDa
anti-FLAG
anti-HA
Ndt80-FLAG + + + +
Ume6-HA + - + +
Ume6^1-720-HA - - + -
Ume6^1-759-HA - - - +
Upc2-FLAG + + + +
Ume6-HA + - + +
Ume6^1-720-HA - - + -
Ume6^1-759-HA - - - +

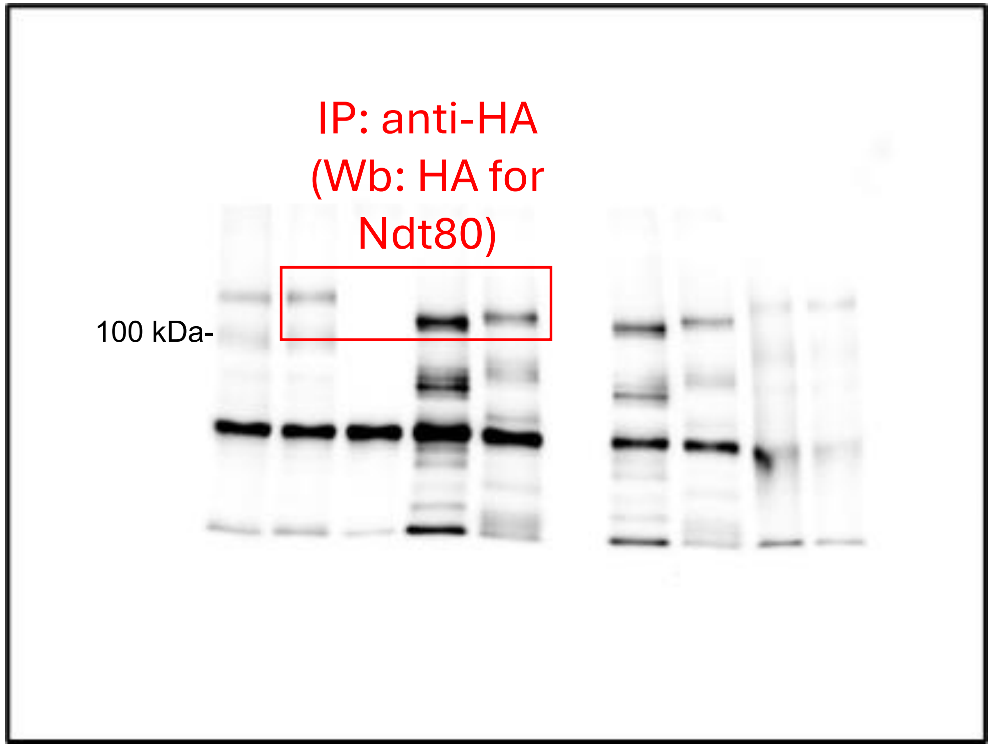
IP: anti-HA
(Wb: HA for
Ndt80)
100 kDa-

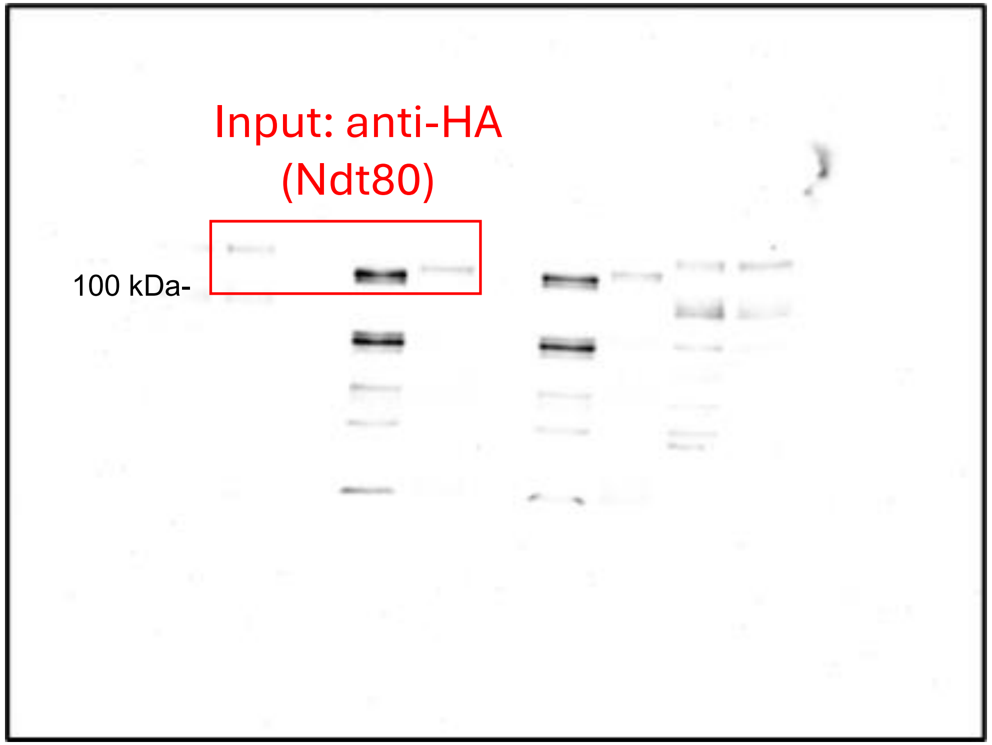
Input: anti-HA
(Ndt80)
100 kDa-

Fig 4d

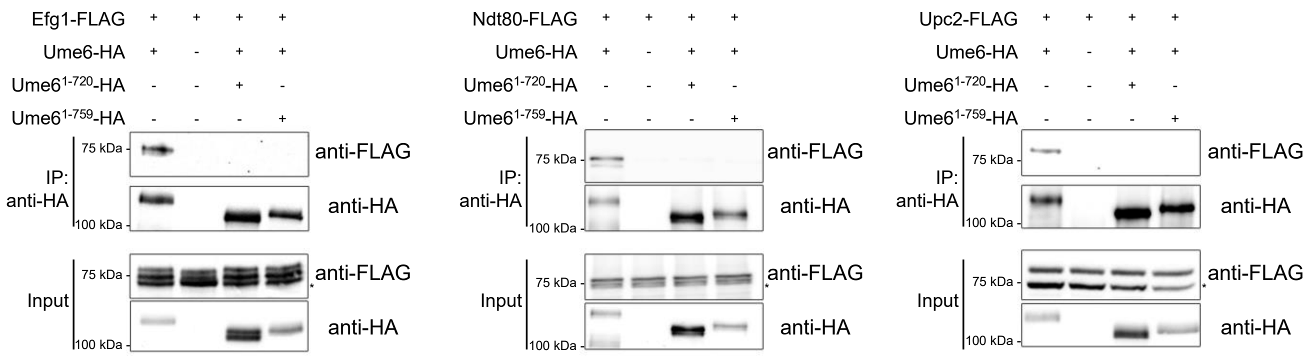
Efg1-FLAG + + + +
Ume6-HA + - + +
Ume6$^{1-720}$-HA - - + -
Ume6$^{1-759}$-HA - - - +
IP: anti-HA
Input
75 kDa
100 kDa
anti-FLAG
anti-HA
Ndt80-FLAG + + + +
Upc2-FLAG + + + +

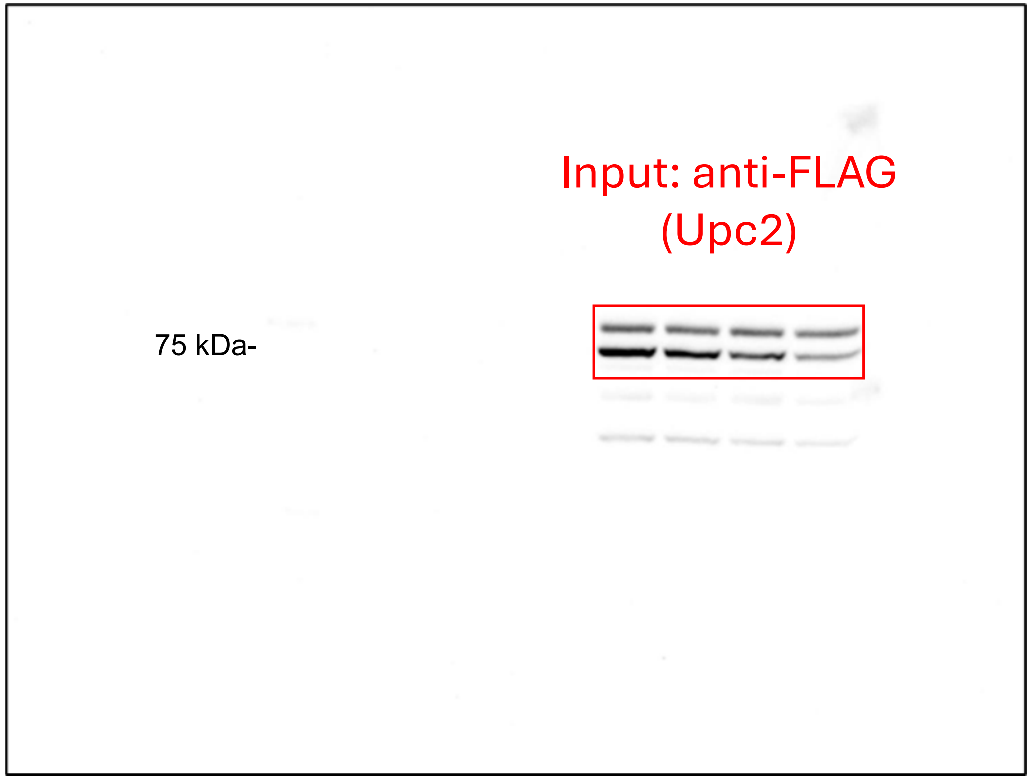
Input: anti-FLAG (Upc2)
75 kDa-

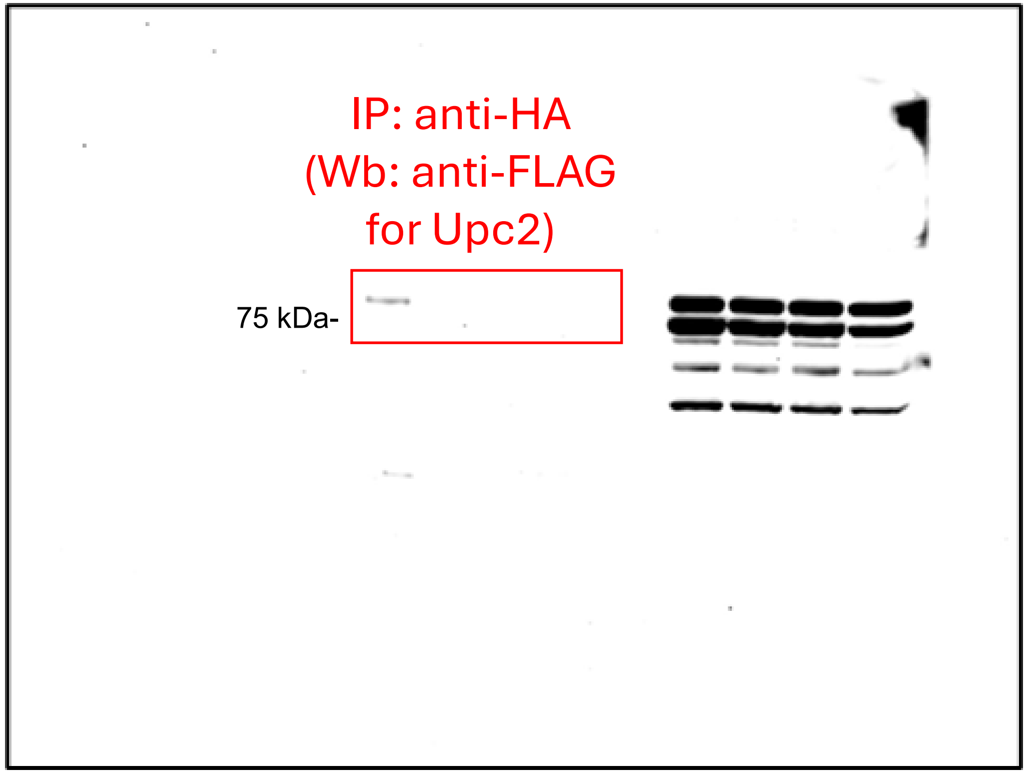
IP: anti-HA (Wb: anti-FLAG for Upc2)
75 kDa-

Fig 4d

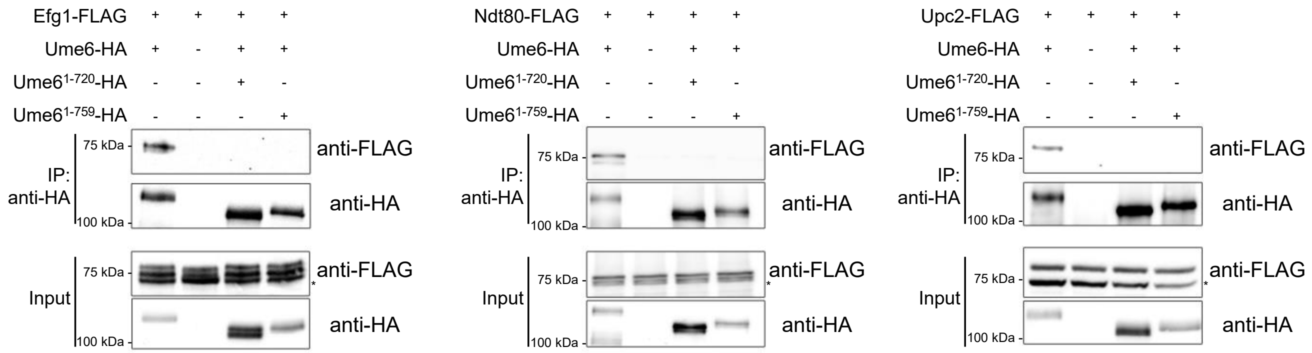

Efg1-FLAG + + + +
Ume6-HA + - + +
Ume6^1-720-HA - - + -
Ume6^1-759-HA - - - +
IP: anti-HA
75 kDa
anti-FLAG
anti-HA
100 kDa
Input
75 kDa
anti-FLAG
anti-HA
100 kDa
Ndt80-FLAG + + + +
Ume6-HA + - + +
Ume6^1-720-HA - - + -
Ume6^1-759-HA - - - +
IP: anti-HA
75 kDa
anti-FLAG
anti-HA
100 kDa
Input
75 kDa
anti-FLAG
anti-HA
100 kDa
Upc2-FLAG + + + +
Ume6-HA + - + +
Ume6^1-720-HA - - + -
Ume6^1-759-HA - - - +
IP: anti-HA
75 kDa
anti-FLAG
anti-HA
100 kDa
Input
75 kDa
anti-FLAG
anti-HA
100 kDa

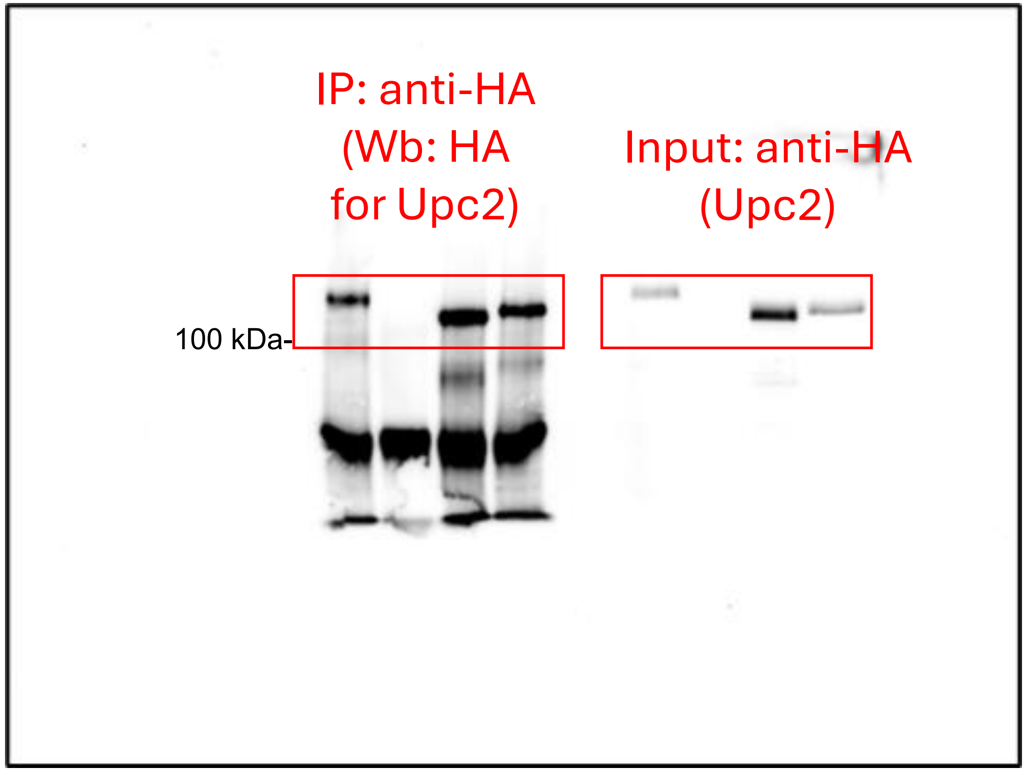

IP: anti-HA
(Wb: HA
for Upc2)
Input: anti-HA
(Upc2)
100 kDa-

Fig 4h

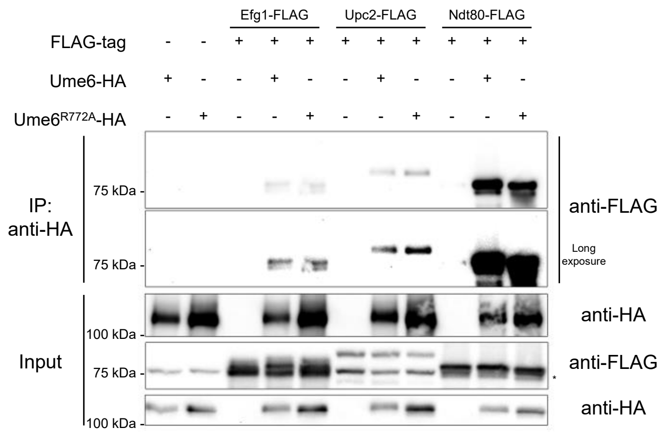
Efg1-FLAG
Upc2-FLAG
Ndt80-FLAG
FLAG-tag
Ume6-HA
Ume6R772A-HA
IP: anti-HA
75 kDa
anti-FLAG
Long exposure
Input
100 kDa
anti-HA

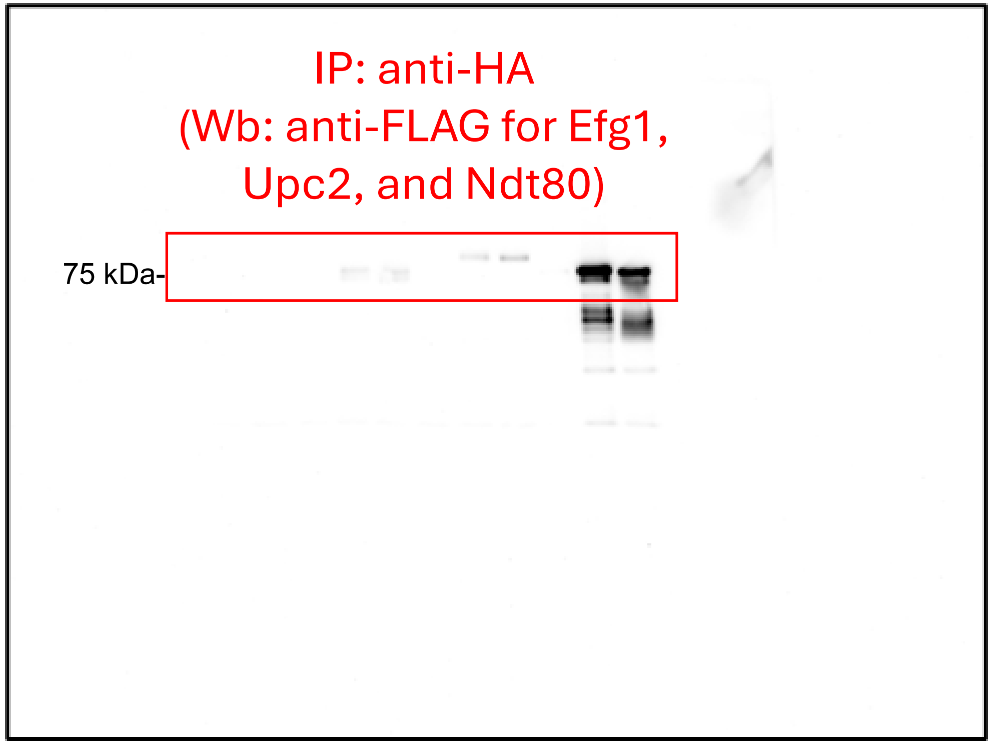
IP: anti-HA
(Wb: anti-FLAG for Efg1, Upc2, and Ndt80)
75 kDa-

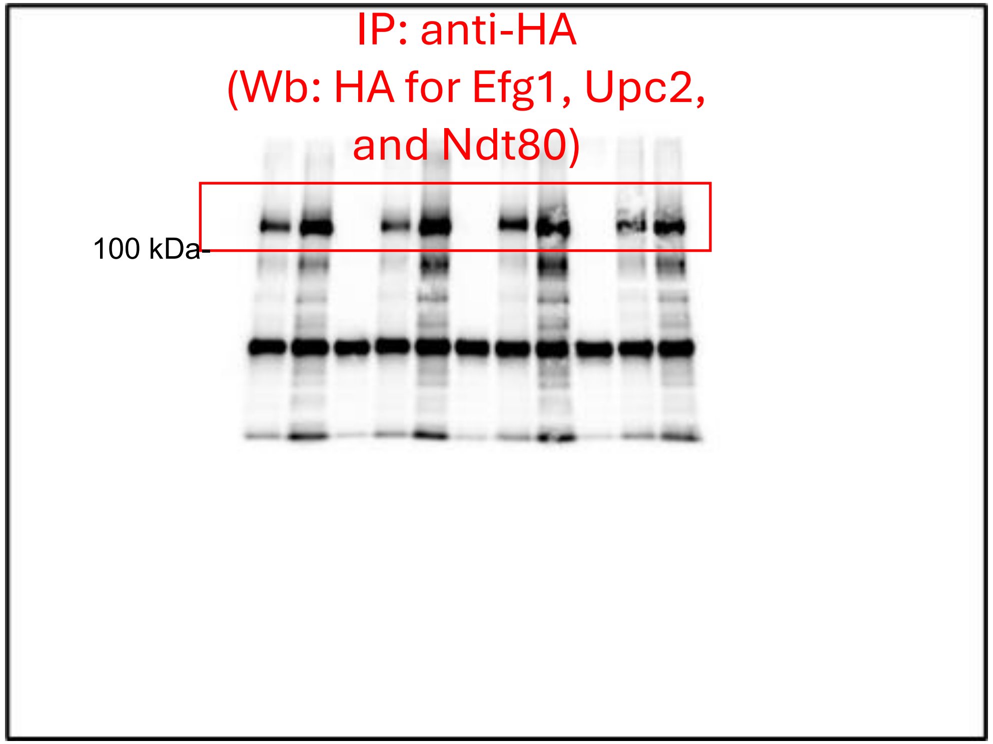
IP: anti-HA
(Wb: HA for Efg1, Upc2, and Ndt80)
100 kDa-

Fig 4h

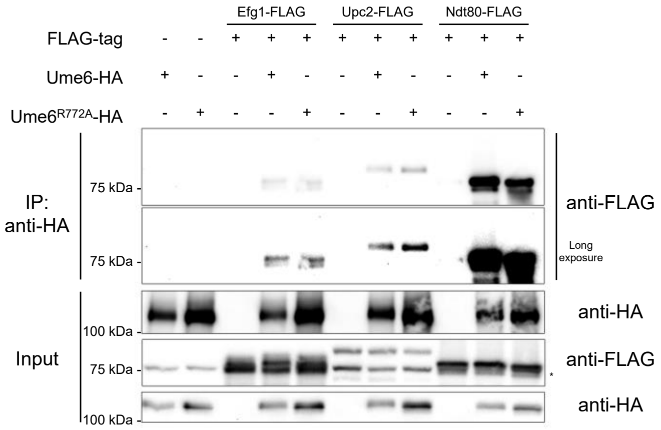
Efg1-FLAG
Upc2-FLAG
Ndt80-FLAG
FLAG-tag
Ume6-HA
Ume6R772A-HA
IP:
anti-HA
75 kDa
75 kDa
anti-FLAG
Long exposure
Input
100 kDa
75 kDa
100 kDa
anti-HA
anti-FLAG
anti-HA

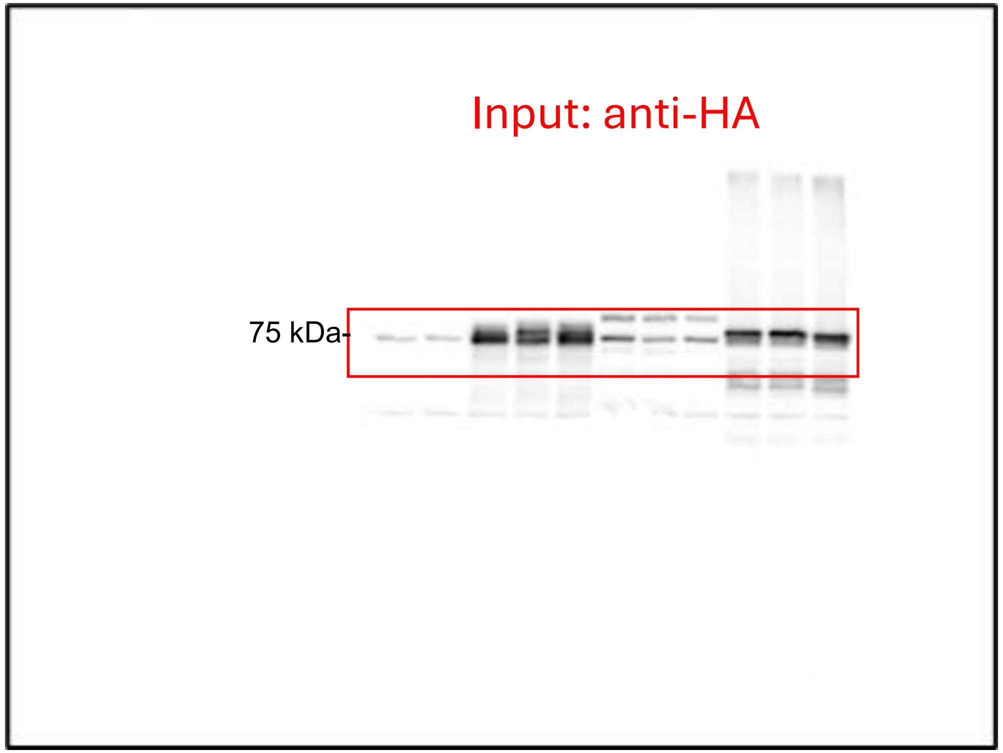
Input: anti-HA
75 kDa-

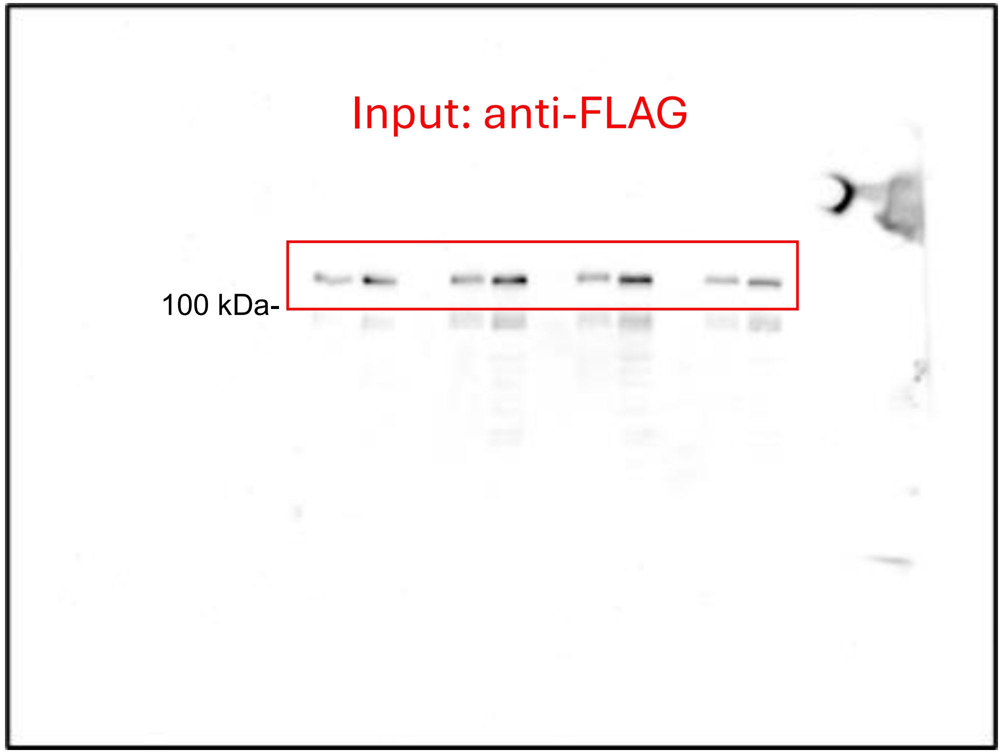
Input: anti-FLAG
100 kDa-

Fig 5d

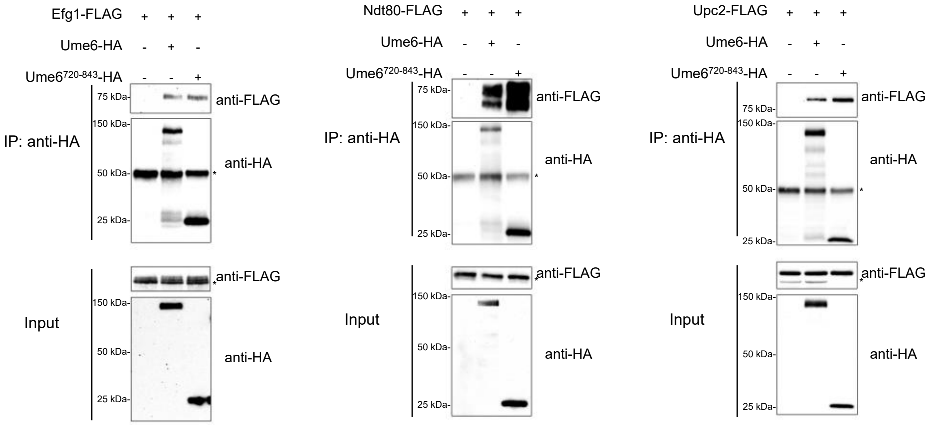

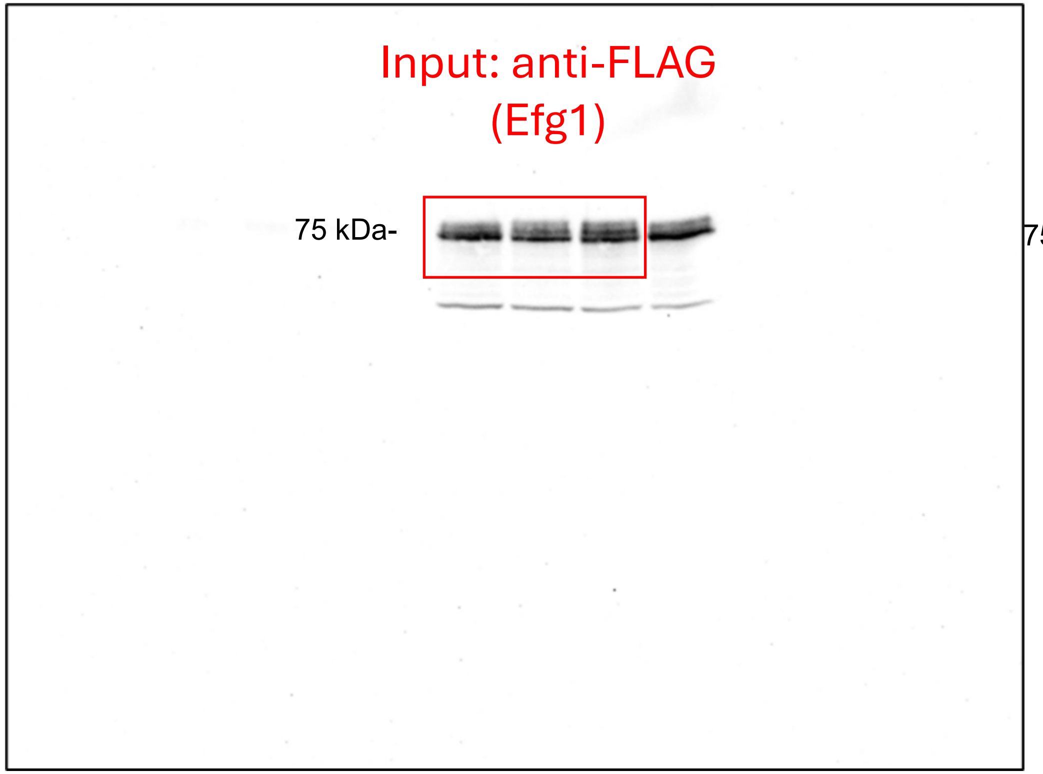

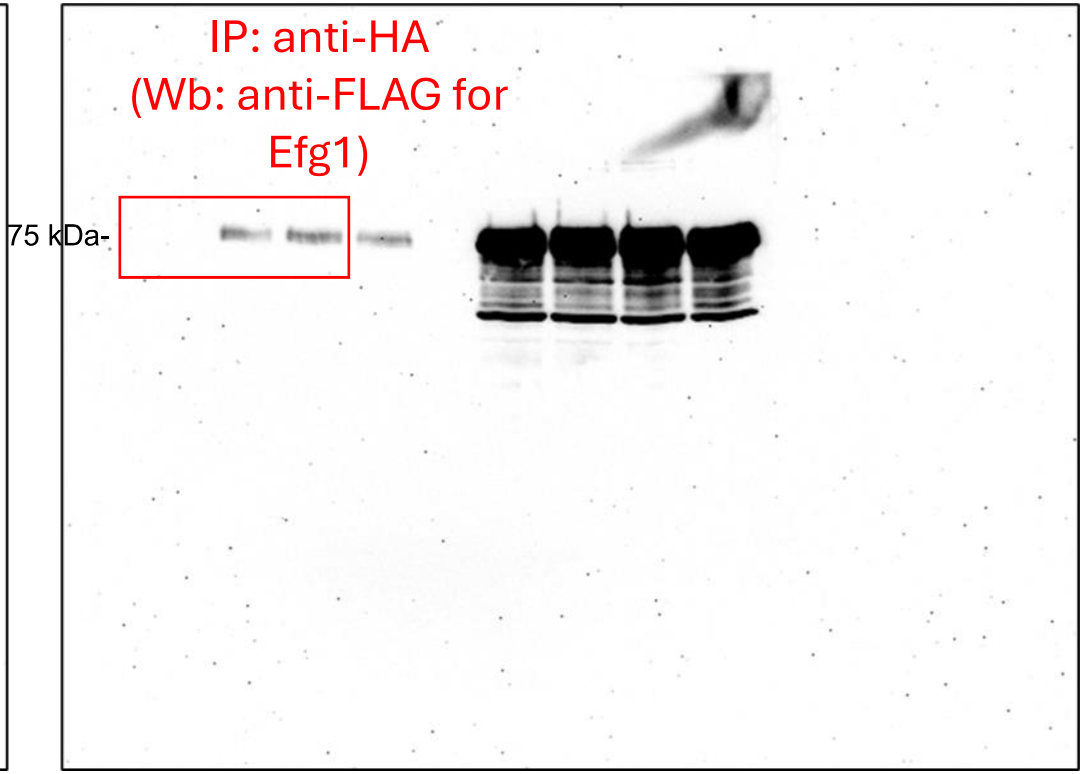

Input: anti-FLAG (720-843) for Efg1

IP: anti-HA (720-843) for Efg1
Long exposure

Fig 5d

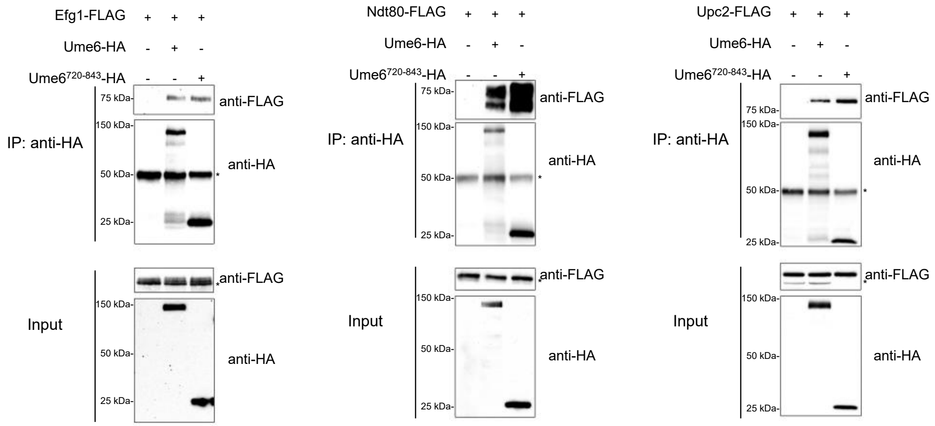
Efg1-FLAG + + +
Ume6-HA - + -
Ume6720-843-HA - - +
IP: anti-HA
Input
anti-FLAG
anti-HA
Ndt80-FLAG + + +
Ume6-HA - + -
Ume6720-843-HA - - +
Upc2-FLAG + + +
Ume6-HA - + -
Ume6720-843-HA - - +
75 kDa-
150 kDa-
50 kDa-
25 kDa-

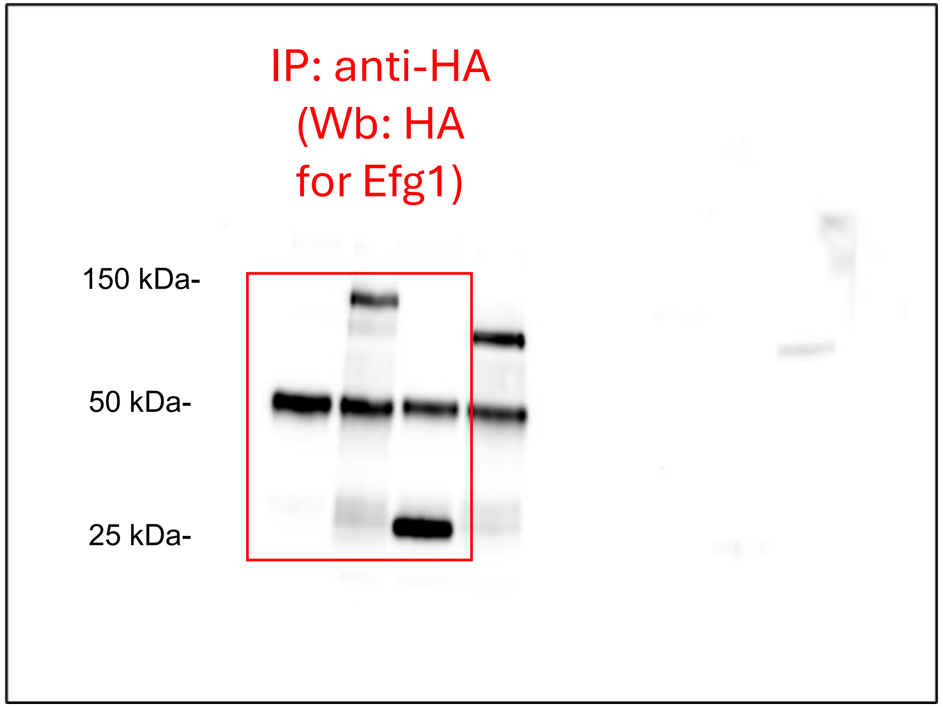
IP: anti-HA
(Wb: HA
for Efg1)
150 kDa-
50 kDa-
25 kDa-

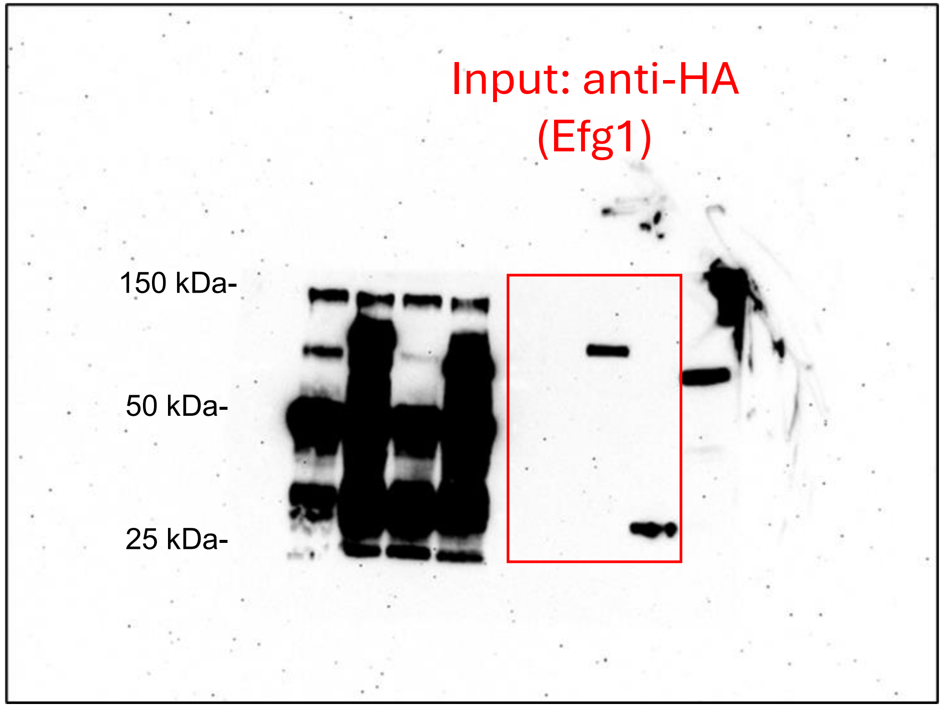
Input: anti-HA
(Efg1)
150 kDa-
50 kDa-
25 kDa-

Fig 5d

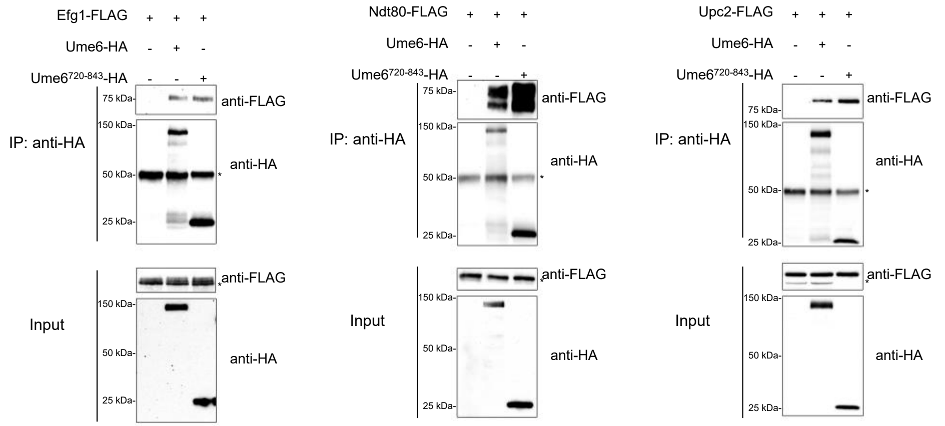

Efg1-FLAG + + +
Ume6-HA - + -
Ume6720-843-HA - - +
IP: anti-HA
Input
anti-FLAG
anti-HA
75 kDa-
150 kDa-
50 kDa-
25 kDa-
Ndt80-FLAG + + +
Ume6-HA - + -
Ume6720-843-HA - - +
Upc2-FLAG + + +
Ume6-HA - + -
Ume6720-843-HA - - +

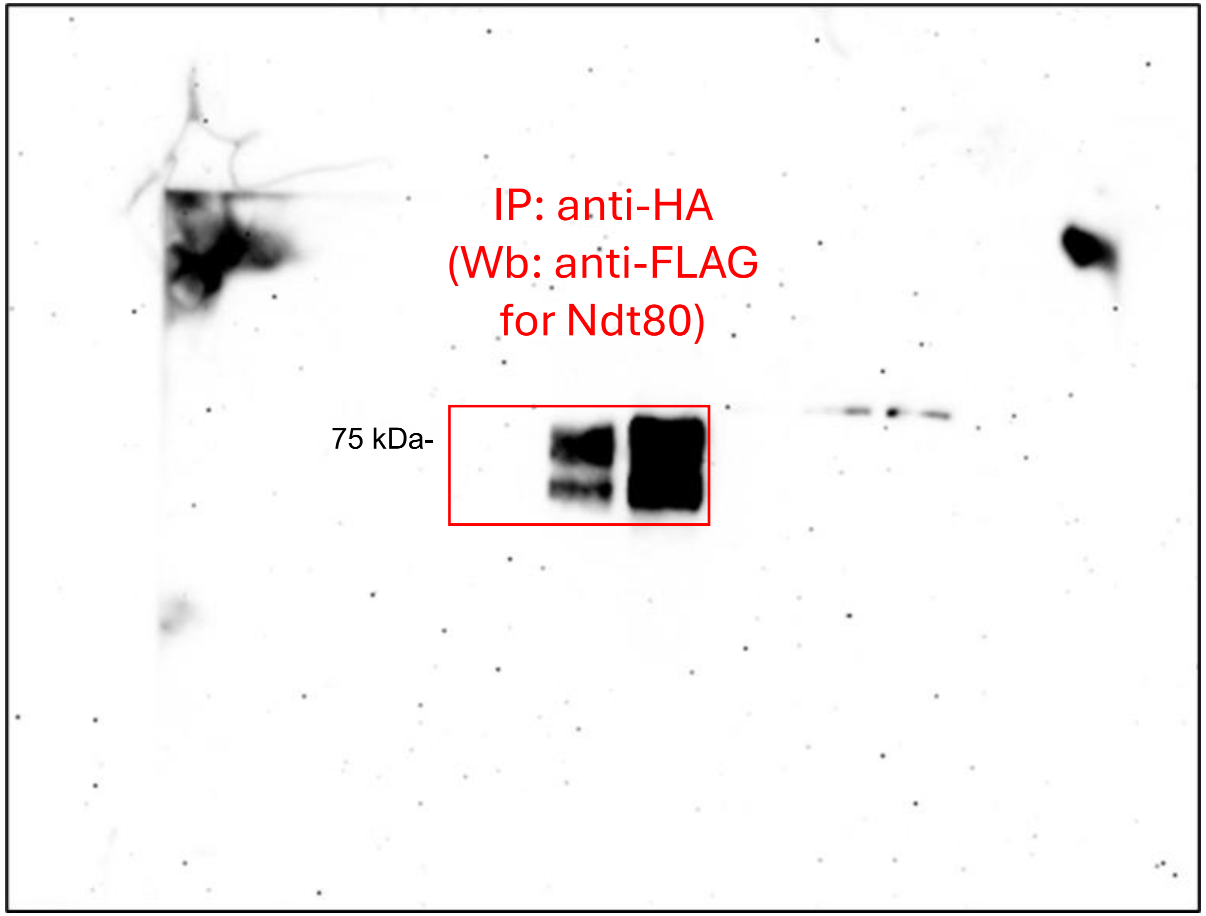

IP: anti-HA
(Wb: anti-FLAG
for Ndt80)
75 kDa-

Fig 5d

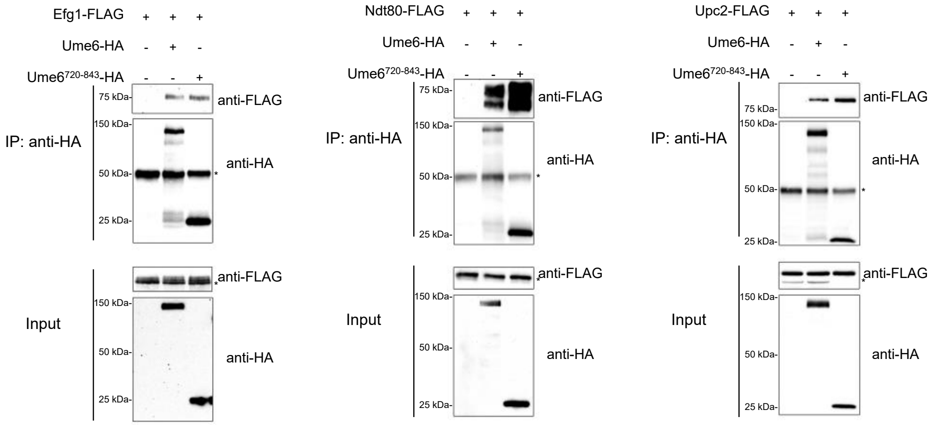

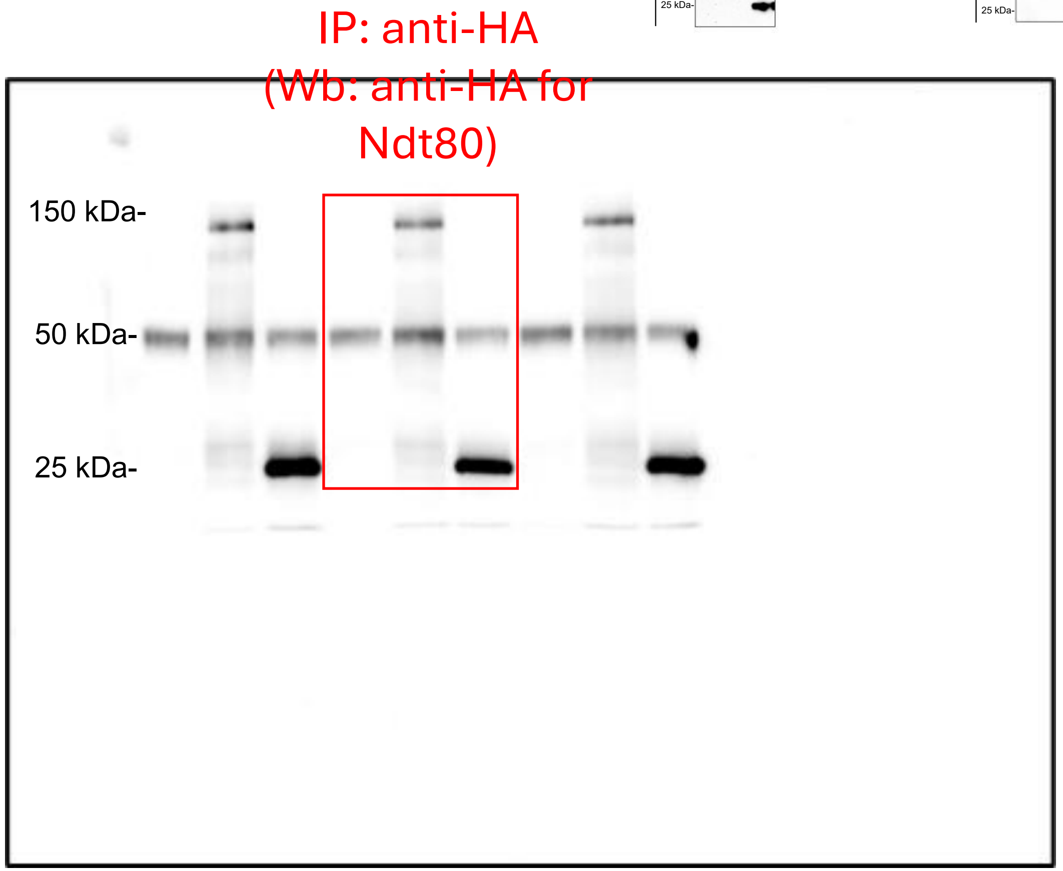

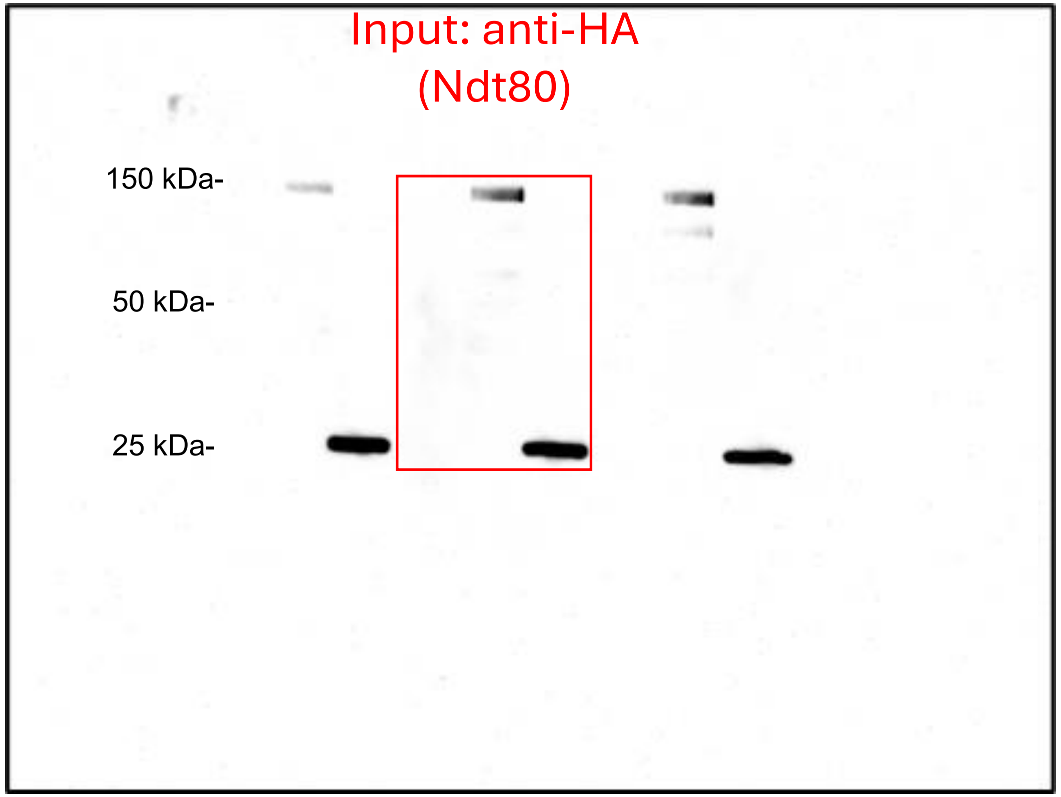

IP: anti-HA (720-843) for Ndt80

Fig 5d

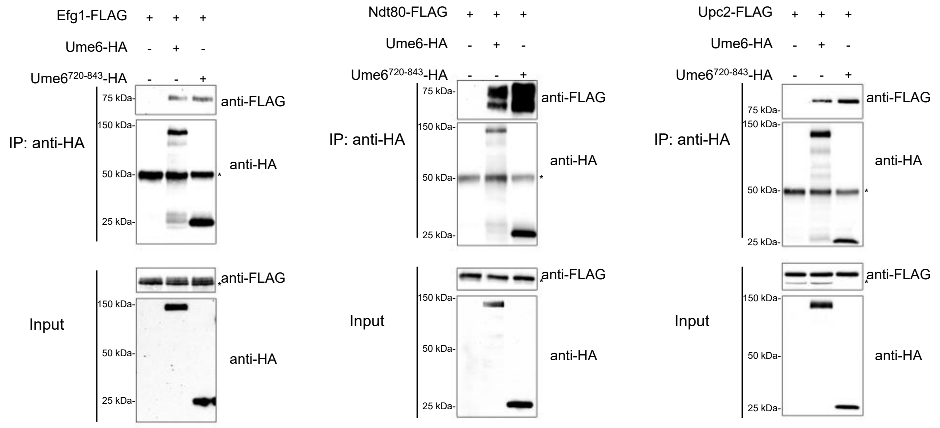
Efg1-FLAG + + +
Ume6-HA - + -
Ume6720-843-HA - - +
IP: anti-HA
anti-FLAG
anti-HA
Input
anti-FLAG
anti-HA
75 kDa-
150 kDa-
50 kDa-
25 kDa-
Ndt80-FLAG + + +
Ume6-HA - + -
Ume6720-843-HA - - +
Upc2-FLAG + + +
Ume6-HA - + -
Ume6720-843-HA - - +

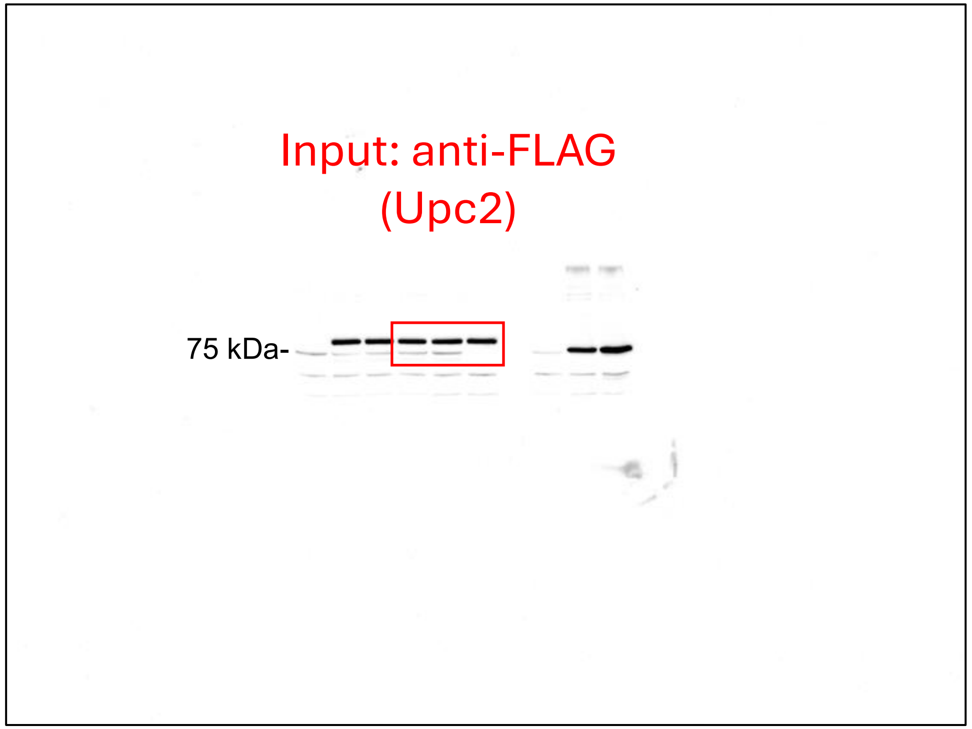
Input: anti-FLAG
(Upc2)
75 kDa-

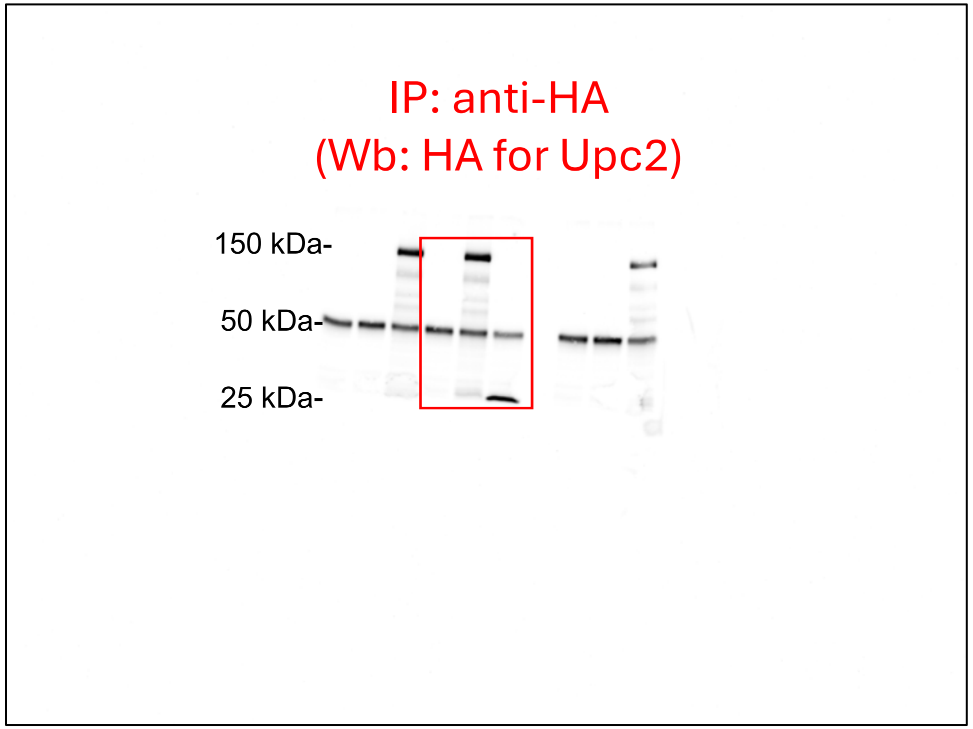
IP: anti-HA
(Wb: HA for Upc2)
150 kDa-
50 kDa-
25 kDa-

Fig 5d

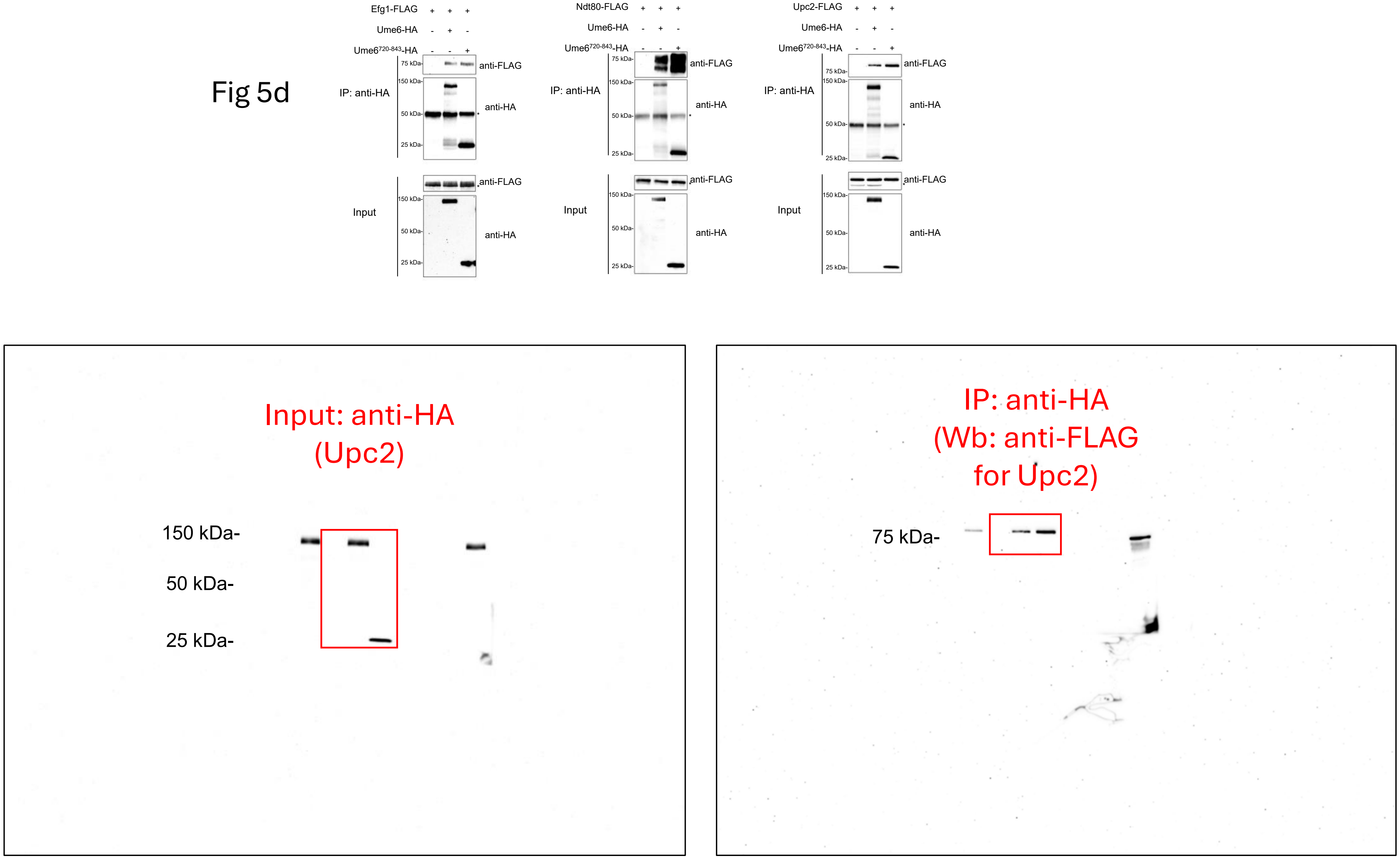
Efg1-FLAG + + +
Ume6-HA - + -
Ume6720-843-HA - - +
IP: anti-HA
anti-FLAG
anti-HA
Input
anti-FLAG
anti-HA
Ndt80-FLAG + + +
Upc2-FLAG + + +
75 kDa-
150 kDa-
50 kDa-
25 kDa-
Input: anti-HA
(Upc2)
IP: anti-HA
(Wb: anti-FLAG
for Upc2)

Fig S1d

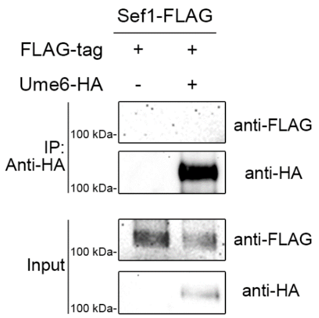
Sef1-FLAG
FLAG-tag + +
Ume6-HA - +
IP: Anti-HA
100 kDa-
anti-FLAG
anti-HA
Input
anti-FLAG
anti-HA

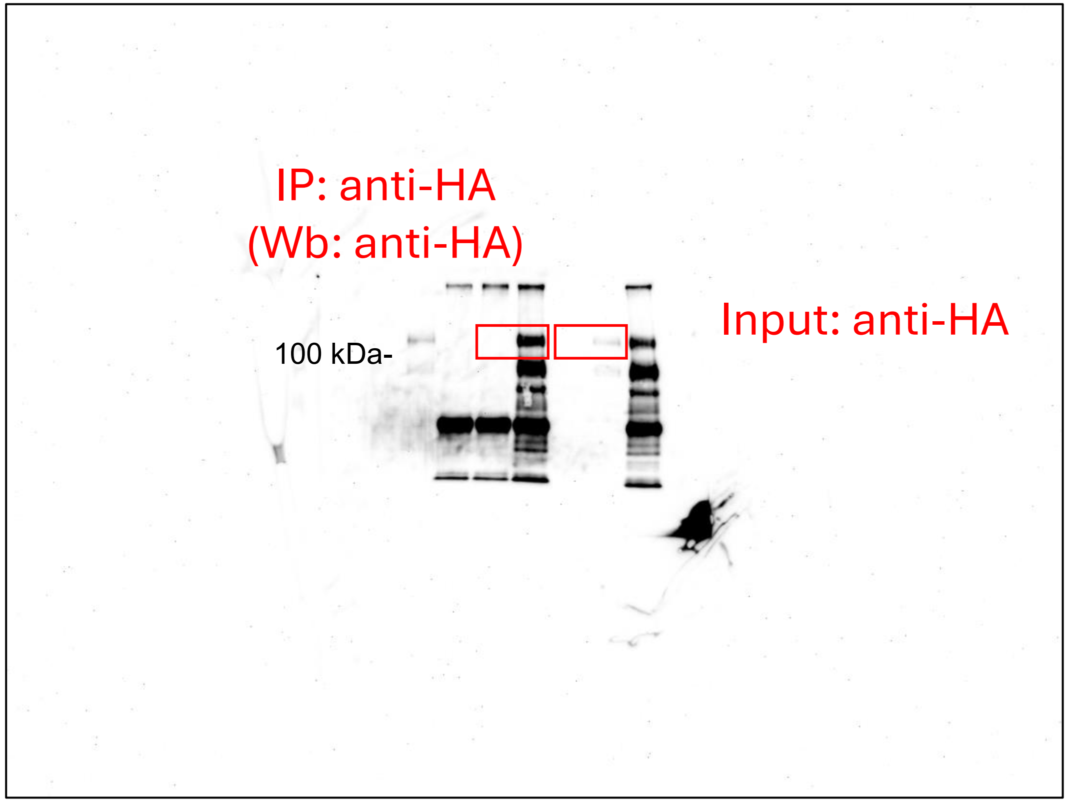
IP: anti-HA
(Wb: anti-HA)
100 kDa-
Input: anti-HA

Fig S1d

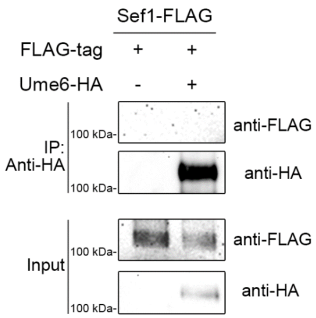
Sef1-FLAG
FLAG-tag + +
Ume6-HA - +
IP: Anti-HA
100 kDa-
anti-FLAG
anti-HA
Input
anti-FLAG
anti-HA

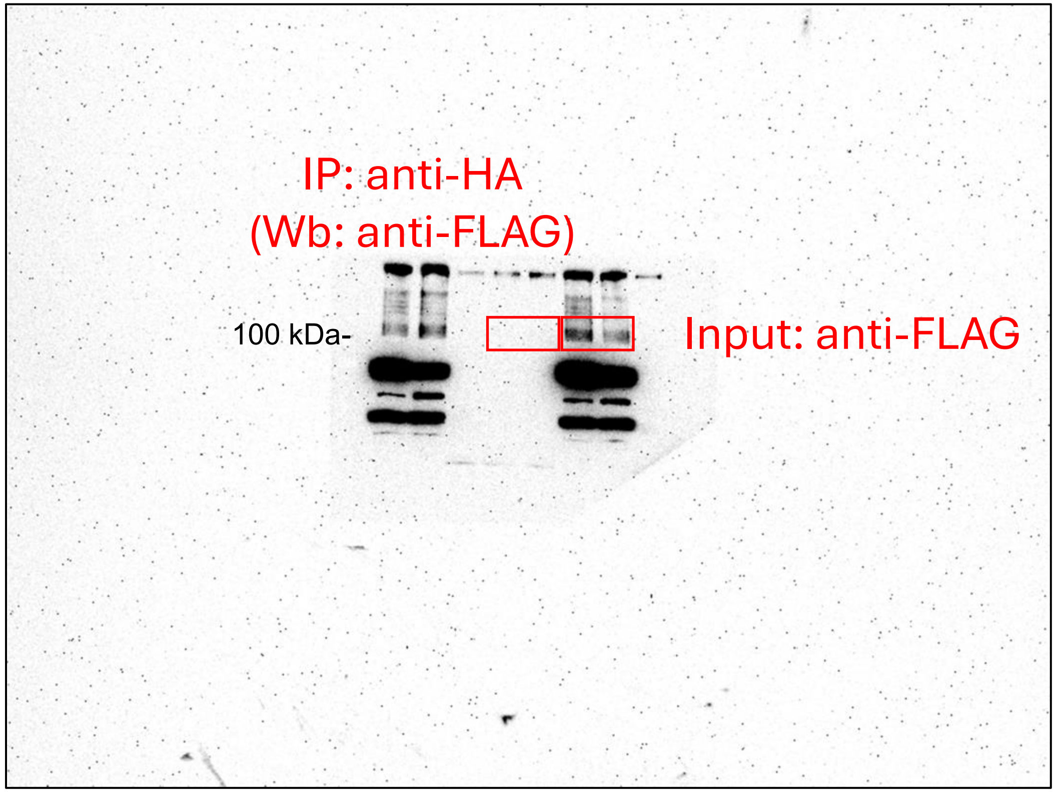
IP: anti-HA
(Wb: anti-FLAG)
100 kDa-
Input: anti-FLAG
